# Nontraditional lipid and lipid-inflammatory parameters for risk stratification of abnormal glucose metabolism: a cross-sectional study in Chinese adults

**DOI:** 10.3389/fendo.2026.1873940

**Published:** 2026-07-10

**Authors:** Dan-jing Chen, Hua Fang, Sheng-gen Wu, Wen Si, Shi-yuan Wang, Li-zhi Liu, Ying Han, Xian-E Peng

**Affiliations:** 1 Department of Epidemiology and Health Statistics, Fujian Provincial Key Laboratory of Environment Factors and Cancer, School of Public Health, Fujian Medical University, Fuzhou, China; 2 Key Laboratory of Gastrointestinal Cancer (Fujian Medical University), Ministry of Education, School of Basic Medical Sciences, Fujian Medical University, Fuzhou, China; 3 Fujian Center for Disease Control and Prevention, Fujian Provincial Key Laboratory of Zoonosis Research, Fuzhou, China; 4 Department of Geriatrics, The First Affiliated Hospital of Fujian Medical University, Fuzhou, China; 5 Fujian Hypertension Research Institute, The First Affiliated Hospital of Fujian Medical University, Fuzhou, China; 6 Branch of National Clinical Research Center for Aging and Medicine, The First Affiliated Hospital of Fujian Medical University, Fuzhou, Fujian, China

**Keywords:** diagnostic ability, dyslipidemia, inflammation, prediabetes, type 2 diabetes

## Abstract

**Objective:**

To evaluate the associations and diagnostic ability of non-traditional lipid parameters and lipid-inflammation parameters with abnormal glucose metabolism, and to investigate the mediating role of high-sensitivity C-reactive protein (hs-CRP).

**Methods:**

Based on cross-sectional survey data from 9,790 adults in Fujian Province, China (2020-2021), 2,301 participants were included in the analysis. Eight non-traditional lipid parameters and lipid-inflammation parameters incorporating hs-CRP were calculated. Associations with impaired glucose metabolism were examined using ordinal logistic regression, restricted cubic spline analysis, and subgroup analyses. Incremental diagnostic ability was evaluated using receiver operating characteristic curve analysis, C-statistics, net reclassification improvement, and integrated discrimination improvement. Mediation analysis was performed to examine the role of hs-CRP.

**Results:**

All parameters were significantly positively associated with impaired glucose metabolism, with several showing nonlinear dose-response relationships. Subgroup analyses demonstrated generally consistent associations with those in the overall population. In the prediabetes comparison, atherogenic coefficient, Castelli’s index-I, remnant cholesterol-C-reactive protein index, and remnant cholesterol to high-density lipoprotein cholesterol-C-reactive protein index (RC/HDL-C-CRP) had superior diagnostic ability, whereas atherogenic index of plasma and RC/HDL-C-CRP performed better in the diabetic comparison. Mediation analysis indicated that hs-CRP partially mediated the association between non-traditional lipid parameters and prediabetes, with bidirectional mediation effects observed. No mediation effect was observed for diabetes risk.

**Conclusion:**

Non-traditional lipid parameters and lipid-inflammation parameters were significantly associated with impaired glucose metabolism. Incorporating inflammatory information may enhance the identification of high-risk individuals, providing early warning particularly in prediabetic populations.

## 1 Introduction

Diabetes is a chronic metabolic disorder that progresses from normal blood glucose metabolism to prediabetes and diabetes (1). Global statistics indicate that approximately 463 million adults worldwide had diabetes in 2019, with projections a rise to 700 million by 2045, posing a substantial burden on global health (2). Furthermore, with increasing severity of diabetes, it often leads to serious complications such as cardiovascular disease, kidney disease, and cancer, resulting in increased mortality (3–5). Currently, diabetes ranks among the top ten causes of death globally (6). In 2019, it contributed to approximately 1.5 million deaths, accounting for 35.6% of all noncommunicable disease deaths and 2.7% of all-cause mortality worldwide (7). Prediabetes, an intermediate stage of glucose dysregulation that often precedes type 2 diabetes, affected approximately 720 million people worldwide in 2021, and this number is projected to reach about 1 billion by 2045 (8). A Chinese population-based cohort study indicates that among participants with prediabetes who did not receive any intervention, the cumulative incidence of diabetes reached 95.9% over a 30-year follow-up period (9). Meanwhile, lifestyle interventions in individuals with impaired glucose tolerance can delay the onset of diabetes, reduce its incidence, and extend life expectancy (9, 10). Therefore, prediabetes as a critical window for intervention, systematically identifying risk factors associated with impaired glucose metabolism, and implementing early screening are crucial. These measures can facilitate the early detection and intervention of the disease, thereby reducing the burden of diabetes and its related complications.

Dyslipidemia plays an important role in the development of abnormal glucose metabolism, and is often observed alongside impaired glucose metabolism (11). The potential mechanism may involve pancreatic cholesterol accumulation by promoting the aggregation of pancreatic amyloid peptides, thereby exacerbating pancreatic amyloidosis, further impairing β-cell function, and disrupting glucose homeostasis (12–14). In recent years, non-traditional lipid parameters calculated from conventional lipid measurements have received growing attention. These parameters are regarded as more accurate in quantifying risk information and better capture the interactions among lipid components (15). Additionally, non-traditional lipid parameters, including the atherosclerosis index of plasma (AIP), atherosclerosis coefficient (AC), and lipoprotein complex index (LCI), have been shown to predict several conditions, such as impaired glucose metabolism, stroke, and non-alcoholic fatty liver disease (16–18). Recently, a large-scale study based on the Chinese adult population showed that several non-traditional lipid parameters are significantly associated with prediabetes and show superior risk discrimination compared with traditional lipid parameters (19). This highlights their potential value in identifying early-stage glucose metabolism abnormalities. However, existing evidence primarily focuses on the prediabetic stage. A systematic evaluation of the role of non-traditional lipid parameters in the overall progression of impaired glucose metabolism remains lacking.

Furthermore, the inflammatory response plays a pivotal role in the pathogenesis and progression of insulin resistance (IR) and contributes to the onset of diabetes (20). High-sensitivity C-reactive protein (hs-CRP) as a commonly used systemic inflammatory marker is closely associated with IR and diabetes risk (21). Moreover, abnormalities in lipid metabolism are closely associated with inflammatory states and synergistically contribute to metabolic disorder processes. Specifically, excessive or abnormal lipid accumulation activates inflammatory signaling, promoting the expression of inflammatory parameters. Conversely, inflammation disrupts lipid metabolism and exacerbates lipid-induced toxicity, thereby worsening IR and metabolic dysfunction (22, 23). Therefore, evaluating the impact of lipid metabolism and inflammatory status on impaired glucose metabolism may provide robust scientific evidence for diabetes risk assessment.

In this study, we systematically evaluated the associations between non-traditional lipid parameters and lipid-inflammation parameters with impaired glucose metabolism in a Chinese adult population. Evaluate their diagnostic ability and incremental diagnostic ability in risk identification, while further exploring the mediating role of hs-CRP in this association. These findings may facilitate the early recognition and risk assessment of impaired glucose metabolism in the population.

## 2 Methods

### 2.1 Study design and population

This study was based on cross-sectional survey data from the Fujian Provincial Monitoring Site of the China Cardiovascular Disease and Risk Factors Surveillance Program, collected between August 2020 and April 2021 (24). This cross-sectional study included a total of 9,790 adult participants who had resided in Fujian Province, China for ≥6 months. Detailed procedures for questionnaire surveys, physical examinations, and laboratory tests are described in the **Supplementary Methods 1**. After excluding participants with (a) missing lipid biomarker data, (b) missing hs-CRP data, and (c) missing data for other relevant covariates, a total of 2,301 participants were included in the final analysis (**Supplementary Figure 1**). To evaluate the potential for selection bias resulting from complete-case analysis, baseline characteristics were compared between included and excluded participants, and the results are presented in **Supplementary Table 1**. All participants provided written informed consent. For those unable to write, fingerprint identification was used. This study was approved by the Medical Research Ethics Committees of Fuwai Hospital, Beijing (Approval No.2020-1360).

### 2.2 Definition of prediabetes and diabetes

Prediabetes was defined as (a) fasting blood glucose (FBG) levels of 6.1-7.0 mmol/L, or (b) glycated hemoglobin (HbA1c) levels of 5.7-6.5%. Diabetes was defined as meeting any of the following criteria: (a) self-reported physician-diagnosed diabetes, confirmed by a positive response to the question: “Has a doctor or other health professional ever told you that you have diabetes?” (b) current use of hypoglycemic medication or insulin; (c) FBG≥7.0 mmol/L; or (d) HbA1c≥6.5% (25).

### 2.3 Calculation of non-traditional lipid and non-traditional lipid-inflammation parameters

Traditional lipid markers, including total cholesterol (TC), triglycerides (TG), high-density lipoprotein cholesterol (HDL-C), and low-density lipoprotein cholesterol (LDL-C), were used to calculate and evaluate eight non-traditional lipid indices, with all definitions and calculation formulas provided in **Supplementary Method 2**. These parameters included AIP, non-HDL-C, AC, Castelli’s index I (CRI-I) and II (CRI-II), LCI, remnant cholesterol (RC); and RC/HDL-C. Additionally, non-traditional lipid-inflammation parameters were calculated by integrating hs-CRP, which is uniformly referred to as CRP throughout this study. For all 16 biomarkers, interquartile range (IQR) standardization was applied to the continuous levels, and the biomarkers were simultaneously categorized into quartiles (Q1-Q4) for categorical analyses. The corresponding quartile cut-off values for each biomarker are provided in **Supplementary Table 2**. These continuous and categorized variables were subsequently used in all further analyses.

### 2.4 Covariates

This study accounted for potential confounding factors associated with prediabetes and diabetes, including age, sex, body mass index (BMI, kg/m²), educational level, marital status, smoking, drinking status, hypertension, and dyslipidemia drug. Educational level was categorized as less than high school, high school and above. Marital status was categorized as married (married or cohabiting with a partner) and other (including unmarried, widowed, divorced, and separated). Based on exposure at the time of the survey, smoking status was classified as current smokers and non-smokers (never smoked or former smokers), and drinking status was categorized as current drinkers and non-drinkers (never drank or former drinkers). Hypertension was defined as a self-reported history of hypertension, systolic blood pressure (SBP)≥140 mmHg and/or diastolic blood pressure (DBP)≥90 mmHg, or current use of antihypertensive medication.

### 2.5 Statistical analysis

The Kruskal-Wallis test and χ² test were used to assess balance for continuous and categorical variables, respectively. Continuous variables were presented as median and IQR, while categorical variables were presented as numbers (percentages). Before performing regression analysis, multicollinearity and correlations among covariates were examined (**Supplementary Method 3**). Although age showed a significant negative correlation with educational attainment (*r* = -0.85), all variance inflation factors (VIF) were below 3, indicating no severe multicollinearity among the covariates (**Supplementary Figures 2**, **3**).

The test of parallel lines was used to assess the proportional odds assumption in the ordinal logistic regression model. The test yielded a *P* value of 0.27, indicating that the proportional odds assumption was satisfied. Ordered logistic regression was employed to assess the associations of non-traditional lipid parameters and non-traditional lipid-inflammation parameters with the risks of prediabetes and diabetes. Three models were employed to further adjust for potential confounding factors. Model 1 was an unadjusted crude model; Model 2 was adjusted for age, sex, BMI, educational level, and marital status; and Model 3 further included smoking, drinking status, hypertension, and dyslipidemia drug. Restricted cubic splines (RCS) were employed to investigate the dose-response relationship between non-traditional lipid parameters and non-traditional lipid-inflammation parameters and the risk of abnormal glucose metabolism. Additionally, the analysis was stratified by age, sex, BMI, smoking, drinking status, and hypertension to further explore these associations in different subgroups.

To evaluate the incremental predictive value of each lipid index across stages of glucose metabolism, the study population was divided into two comparison settings: (a) normal glycemia versus prediabetes, and (b) non-diabetes (normal glycemia plus prediabetes) versus diabetes. Within each comparison setting, receiver operating characteristic (ROC) curve analysis was performed to calculate the area under the curve (AUC), optimal cutoff value, sensitivity, and specificity. The incremental diagnostic performance of each lipid index beyond the baseline model was assessed using the C-statistic, net reclassification improvement (NRI), and integrated discrimination improvement (IDI). Mediation analysis was conducted using the “mediation” package to examine whether hs-CRP mediates the associations between non-traditional lipid parameters and the risk of abnormal glucose metabolism.

All statistical analyses were performed using R software (version 4.5.1), and graphical visualizations were generated using the “ggplot2” package. A two-sided *P* value<0.05 was considered statistically significant.

## 3 Results

### 3.1 General characteristics of participants

This study included 2,301 participants with a median age of 43 (30, 58) years, including 1,393 with normal glycemia, 708 with prediabetes, and 200 with diabetes (**Table 1**). There was no significant difference in sex distribution among the three groups (*P* = 0.700). Compared with participants with normal glycemia, those with prediabetes or diabetes were older and had higher BMI, and were more likely to have lower educational level, a higher proportion of unmarried, widowed, divorced, or separated individuals, more current smokers and non-drinkers, and a greater likelihood of lipid-lowering medication users (all *P* < 0.05).

**Table 1:** Baseline characteristics of all participants.

Variables	Overall	Non-diabetes	Pre-diabetes	Diabetes	P-value
	(n=2301)	(n=1393)	(n=708)	(n=200)	
Age (years)	43 (30, 58)	35 (25, 47)	53 (44, 67)	60 (51, 70)	<0.001
Sex, n (%)					0.7
Male	1,129 (49.07%)	677 (48.60%)	356 (50.28%)	96 (48.00%)	
Female	1,172 (50.93%)	716 (51.40%)	352 (49.72%)	104 (52.00%)	
Age group, n (%)					<0.001
<60 years	1,770 (76.92%)	1,216 (87.29%)	456 (64.41%)	98 (49.00%)	
≥60 years	531 (23.08%)	177 (12.71%)	252 (35.59%)	102 (51.00%)	
BMI (kg/m2)	23.8 (21.3, 26.4)	23.0 (20.7, 25.4)	24.7 (22.6, 27.1)	26.1 (23.5, 28.4)	<0.001
Educational level, n (%)					<0.001
Less than high school	1,221 (53.06%)	560 (40.20%)	502 (70.90%)	159 (79.50%)	
High school and above	1,080 (46.94%)	833 (59.80%)	206 (29.10%)	41 (20.50%)	
Marital status, n (%)					<0.001
Married/Living with partner	652 (28.34%)	512 (36.76%)	110 (15.54%)	30 (15.00%)	
Others	1,649 (71.66%)	881 (63.24%)	598 (84.46%)	170 (85.00%)	
Smoking status, n (%)					<0.001
Yes	514 (22.34%)	263 (18.88%)	193 (27.26%)	58 (29.00%)	
No	1,787 (77.66%)	1,130 (81.12%)	515 (72.74%)	142 (71.00%)	
Drinking status, n (%)					0.044
Yes	705 (30.64%)	453 (32.52%)	200 (28.25%)	52 (26.00%)	
No	1,596 (69.36%)	940 (67.48%)	508 (71.75%)	148 (74.00%)	
Hypertension, n (%)					<0.001
Yes	705 (30.64%)	263 (18.88%)	307 (43.36%)	135 (67.50%)	
No	1,596 (69.36%)	1130(81.12%)	401(56.64%)	65(32.50%)	
Dyslipidemia drug, n (%)					<0.001
Yes	18 (0.78%)	3 (0.22%)	6 (0.85%)	9 (4.50%)	
No	2,283 (99.22%)	1,390 (99.78%)	702 (99.15%)	191 (95.50%)	

Significant differences were also observed in traditional lipid parameters, non-traditional lipid parameters, and lipid-inflammation parameters across the three groups (**Supplementary Table 3**). Compared with the normal glycemia group, participants with prediabetes or diabetes had higher levels of TG, TC, LDL-C, hs-CRP, non-traditional lipid parameters, and lipid-inflammation parameters, but lower HDL-C (*P* < 0.001).

### 3.2 Association of non-traditional lipid parameters with abnormal glucose metabolism

Ordinal logistic regression was used to analyze the association between eight non-traditional lipid parameters and glucose metabolism status (**Table 2**). The analysis indicated that elevated levels of non-traditional lipid parameters were associated with increased risk of glucose metabolism abnormalities. In the fully adjusted model, each IQR increase in non-traditional lipid parameters was associated with a 1.317-2.002 times higher risk of being in a more severe category of glucose metabolism abnormalities. Similar results were observed when non-traditional lipid parameters were analyzed as categorical variables. Compared with the lowest quartile (Q1), participants in the highest quartile (Q4) had a 2.381-4.516 times higher risk of being in a more severe category of glucose metabolism abnormalities.

**Table 2:** Association analysis between non-traditional lipid parameters and diabetic status.

	Model 1		Model 2		Model 3	
	*OR* (95%*CI*)	*P*	*OR* (95%*CI*)	*P*	*OR* (95%*CI*)	*P*
AIP						
Continuous	2.439(2.175,2.736)	<0.001	2.089(1.820,2.399)	<0.001	2.002(1.741,2.302)	<0.001
Q1						
Q2	2.118(1.625,2.761)	<0.001	1.641(1.222,2.203)	0.001	1.611(1.198,2.166)	0.002
Q3	2.980(2.299,3.862)	<0.001	1.698(1.264,2.280)	<0.001	1.681(1.251,2.259)	0.001
Q4	6.293(4.858,8.151)	<0.001	3.946(2.912,5.348)	<0.001	3.706(2.730,5.033)	<0.001
P for trend	<0.001		<0.001		<0.001	
Non-HDL-C						
Continuous	2.345(2.088,2.633)	<0.001	1.618(1.428,1.834)	<0.001	1.615(1.424,1.832)	<0.001
Q1						
Q2	1.473(1.129,1.923)	0.004	1.018(0.759,1.364)	0.908	1.059(0.788,1.424)	0.703
Q3	2.879(2.230,3.716)	<0.001	1.572(1.185,2.086)	0.002	1.648(1.238,2.193)	0.001
Q4	5.432(4.220,6.993)	<0.001	2.318(1.754,3.063)	<0.001	2.381(1.796,3.157)	<0.001
P for trend	<0.001		<0.001		<0.001	
AC						
Continuous	2.642(2.355,2.962)	<0.001	1.981(1.741,2.254)	<0.001	1.949(1.710,2.221)	<0.001
Q1						
Q2	2.428(1.835,3.213)	<0.001	1.793(1.320,2.435)	<0.001	1.903(1.396,2.595)	<0.001
Q3	3.928(2.993,5.156)	<0.001	2.323(1.716,3.143)	<0.001	2.423(1.784,3.290)	<0.001
Q4	8.657(6.600,11.354)	<0.001	4.434(3.263,6.024)	<0.001	4.516(3.311,6.160)	<0.001
P for trend	<0.001		<0.001		<0.001	
CRI-I						
Continuous	2.641(2.355,2.962)	<0.001	1.981(1.741,2.254)	<0.001	1.949(1.710,2.221)	<0.001
Q1						
Q2	2.428(1.835,3.213)	<0.001	1.793(1320,2.435)	<0.001	1.903(1.396,2.595)	<0.001
Q3	3.928(2.993,5.156)	<0.001	2.323(1.716,3.143)	<0.001	2.423(1.784,3.290)	<0.001
Q4	8.657(6.600,11.354)	<0.001	4.434(3.263,6.024)	<0.001	4.516(3.311,6.160)	<0.001
P for trend	<0.001		<0.001		<0.001	
CRI-II						
Continuous	2.285(2.035,2.567)	<0.001	1.673(1.468,1.906)	<0.001	1.689(1.480,1.929)	<0.001
Q1						
Q2	2.387(1.818,3.133)	<0.001	1.931(1.432,2.604)	<0.001	2.006(1.483,2.712)	<0.001
Q3	3.475(2.664,4.533)	<0.001	2.212(1.644,2.976)	<0.001	2.332(1.727,3.149)	<0.001
Q4	6.535(5.021,8.505)	<0.001	3.357(2.492,4.521)	<0.001	3.516(2.599,4.757)	<0.001
P for trend	<0.001		<0.001		<0.001	
LCI						
Continuous	1.528(1.425,1.639)	<0.001	1.343(1.250,1.443)	<0.001	1.317(1.226,1.415)	<0.001
Q1						
Q2	2.001(1.519,2.636)	<0.001	1.362(1.004,1.847)	0.047	1.435(1.055,1.952)	0.022
Q3	3.689(2.827,4.813)	<0.001	1.902(1.411,2.564)	<0.001	1.982(1.466,2.678)	<0.001
Q4	7.833(6.002,10.222)	<0.001	3.807(2.808,5.161)	<0.001	3.748(2.756,5.098)	<0.001
P for trend	<0.001		<0.001		<0.001	
RC						
Continuous	1.852(1.703,2.014)	<0.001	1.524(1.398,1.662)	<0.001	1.494(1.369,1.631)	<0.001
Q1						
Q2	2.283(1.739,2.9980	<0.001	1.617(1.200,2.178)	0.002	1.633(1.211,2.203)	0.001
Q3	3.749(2.872,4.893)	<0.001	2.073(1.549,2.775)	<0.001	2.062(1.538,2.763)	<0.001
Q4	7.632(5.858,9.944)	<0.001	3.640(2.731,4.851)	<0.001	3.508(2.629,4.684)	<0.001
P for trend	<0.001		<0.001		<0.001	
RC/HDL-C						
Continuous	1.723(1.597,1.859)	<0.001	1.466(1.357,1.584)	<0.001	1.438(1.330,1.555)	<0.001
Q1						
Q2	2.190(1.660,2.889)	<0.001	1.475(1.089,1.998)	0.012	1.506(1.110,2.043)	0.009
Q3	3.590(2.743,4.698)	<0.001	2.024(1.508,2.717)	<0.001	2.038(1.515,2.741)	<0.001
Q4	8.787(6.720,11.489)	<0.001	4.212(3.141,5.647)	<0.001	4.105(3.056,5.515)	<0.001
P for trend	<0.001		<0.001		<0.001	

Model 1: Unadjusted crude model. Model 2: Adjusted by age, sex, BMI, education level and marital status. Model 3: Adjusted by age, sex, BMI, education level, marital status, smoking, drinking status, hypertension, and dyslipidemia drug. AIP, atherogenic index of plasma; Non-HDL-C, non-high-density lipoprotein cholesterol; AC, atherogenic coefficient; CRI-I, Castelli Risk Index I; CRI-II, Castelli Risk Index II; LCI, lipoprotein combined index; RC, remnant cholesterol; RC/HDL-C, remnant cholesterol/high-density lipoprotein cholesterol.

### 3.3 Association of non-traditional lipid-inflammatory parameters with abnormal glucose metabolism

Simultaneously, ordinal logistic regression was used to analyze the association between eight non-traditional lipid-inflammation parameters and glucose metabolism abnormalities (**Table 3**). Results demonstrated that elevated levels of non-traditional lipid-inflammation parameters were significantly associated with increased severity of glucose metabolism abnormalities. In the fully adjusted model, each IQR increase in non-traditional lipid-inflammation parameter levels was associated with 1.057 to 1.154 times higher risk of being in a higher category of glucose metabolism abnormality. When non-traditional lipid-inflammation parameters were analyzed as categorical variables, consistent trends were observed. Compared with Q1, Q4 exhibited 2.614 to 4.206 times higher risk of being in a higher category of glucose metabolism abnormality.

**Table 3:** Association analysis between non-traditional lipid-inflammatory parameters and diabetic status.

	Model 1		Model 2		Model 3	
	*OR* (95%*CI*)	*P*	*OR* (95%*CI*)	*P*	*OR* (95%*CI*)	*P*
AIP-CRP						
Continuous	1.214(1.164,1.267)	<0.001	1.169(1.120,1.221)	<0.001	1.154(1.106,1.205)	<0.001
Q1						
Q2	0.856(0.668,1.098)	0.222	0.945(0.717,1.246)	0.689	0.939(0.711,1.239)	0.654
Q3	1.138(0.894,1.449)	0.293	1.135(0.868,1.485)	0.355	1.095(0.835,1.434)	0.513
Q4	3.514(2.782,4.440)	<0.001	2.820(2.158,3.686)	<0.001	2.614(1.995,3.426)	<0.001
P for trend	<0.001		<0.001		<0.001	
Non-HDL-C-CRP						
Continuous	1.209(1.148,1.274)	<0.001	1.065(1.013,1.120)	0.013	1.057(1.006,1.111)	0.028
Q1						
Q2	2.335(1.771,3.077)	<0.001	1.492(1.104,2.018)	0.009	1.593(1.175,2.160)	0.003
Q3	3.952(3.020,5.171)	<0.001	2.087(1.549,2.814)	<0.001	2.227(1.648,3.010)	<0.001
Q4	7.245(5.544,9.467)	<0.001	3.081(2.282,4.159)	<0.001	3.101(2.292,4.197)	<0.001
P for trend	<0.001		<0.001		<0.001	
AC-CRP						
Continuous	1.233(1.171,1.299)	<0.001	1.094(1.039,1.151)	0.001	1.083(1.029,1.140)	0.002
Q1						
Q2	2.544(1.929,3.356)	<0.001	1.647(1.217,2.228)	0.001	1.728(1.274,2.343)	<0.001
Q3	4.103(3.131,5.377)	<0.001	2.185(1.617,2.952)	<0.001	2.293(1.693,3.106)	<0.001
Q4	7.225(5.524,9.451)	<0.001	3.140(2.316,4.258)	<0.001	3.143(2.3112,4.273)	<0.001
P for trend	<0.001		<0.001		<0.001	
CRI-I-CRP						
Continuous	1.219(1.157,1.284)	<0.001	1.083(1.028,1.140)	0.003	1.073(1.019,1.129)	0.007
Q1						
Q2	2.568(1.949,3.384)	<0.001	1.646(1.218,2.224)	0.001	1.737(1.282,2.352)	<0.001
Q3	4.030(3.078,5.276)	<0.001	2.154(1.596,2.908)	<0.001	2.260(1.670,3.058)	<0.001
Q4	6.958(5.323,9.096)	<0.001	3.042(2.247,4.118)	<0.001	3.050(2.248,4.139)	<0.001
P for trend	<0.001		<0.001		<0.001	
CRI-II-CRP						
Continuous	1.204(1.144,1.267)	<0.001	1.070(1.017,1.125)	0.009	1.062(1.010,1.116)	0.018
Q1						
Q2	2.647(2.010,3.485)	<0.001	1.746(1.294,2.358)	<0.001	1.845(1.364,2.497)	<0.001
Q3	3.894(2.973,5.100)	<0.001	2.141(1.587,2.888)	<0.001	2.272(1.679,3.074)	<0.001
Q4	6.872(5.258,8.983)	<0.001	2.986(2.205,4.045)	<0.001	3.019(2.223,4.102)	<0.001
P for trend	<0.001		<0.001		<0.001	
LCI-CRP						
Continuous	1.186(1.141,1.234)	<0.001	1.100(1.062,1.138)	<0.001	1.090(1.053,1.128)	<0.001
Q1						
Q2	1.942(1.470,2.565)	<0.001	1.254(0.924,1.702)	0.146	1.328(0.976,1.806)	0.071
Q3	3.884(2.973,5.075)	<0.001	1.940(1.438,2.618)	<0.001	2.039(1.508,2.756)	<0.001
Q4	8.097(6.198,10.578)	<0.001	3.562(2.622,4.840)	<0.001	3.509(2.577,4.777)	<0.001
P for trend	<0.001		<0.001		<0.001	
RC-CRP						
Continuous	1.246(1.190,1.305)	<0.001	1.130(1.084,1.178)	<0.001	1.115(1.070,1.163)	<0.001
Q1						
Q2	2.359(1.771,3.142)	<0.001	1.585(1.162,2.162)	0.004	1.667(1.219,2.278)	0.001
Q3	4.590(3.485,6.046)	<0.001	2.277(1.684,3.079)	<0.001	2.380(1.756,3.226)	<0.001
Q4	10.188(7.732,13.423)	<0.001	4.281(3.157,5.805)	<0.001	4.206(3.095,5.716)	<0.001
P for trend	<0.001		<0.001		<0.001	
RC/HDL-C-CRP						
Continuous	1.218(1.171,1.267)	<0.001	1.127(1.086,1.170)	<0.001	1.114(1.073,1.156)	<0.001
Q1						
Q2	2.541(1.905,3.389)	<0.001	1.630(1.193,2.227)	0.002	1.695(1.239,2.321)	0.001
Q3	4.839(3.666,6.388)	<0.001	2.390(1.762,3.242)	<0.001	2.506(1.842,3.408)	<0.001
Q4	10.014(7.582,13.227)	<0.001	4.291(3.148,5.849)	<0.001	4.198(3.073,5.737)	<0.001
P for trend	<0.001		<0.001		<0.001	

Model 1: Unadjusted crude model. Model 2: Adjusted by age, sex, BMI, education level and marital status. Model 3: Adjusted by age, sex, BMI, education level, marital status, smoking, drinking status, hypertension, and dyslipidemia drug. CRP refers to high-sensitivity C-reactive protein (hs-CRP) throughout this study; AIP, atherogenic index of plasma; Non-HDL-C, non-high-density lipoprotein cholesterol; AC, atherogenic coefficient; CRI-I, Castelli Risk Index I; CRI-II, Castelli Risk Index II; LCI, lipoprotein combined index; RC, remnant cholesterol; RC/HDL-C, remnant cholesterol/high-density lipoprotein cholesterol.

### 3.4 RCS analysis of non-traditional lipid and lipid-inflammation parameters in relation to glucose metabolism abnormalities

As shown in **Supplementary Figure 4**, RCS analysis was performed to assess the dose-response relationship between non-traditional lipid parameters and glucose metabolism abnormalities. Results indicated that higher levels of non-traditional lipid parameters were associated with increased risk of glucose metabolism abnormalities. Specifically, AC, CRI-I, CRI-II, LCI, RC, and RC/HDL-C exhibited significant nonlinear dose-response relationships (all *P* for nonlinearity<0.05), whereas no nonlinear relationship was observed for AIP and Non-HDL-C (all *P* for nonlinearity>0.05). Furthermore, as shown in **Supplementary Figure 5**, levels of eight non-traditional lipid-inflammation parameters demonstrated nonlinear dose-response relationships with glucose metabolism abnormalities (all *P* for nonlinearity<0.05).

### 3.5 Subgroup analysis of the associations between non-traditional lipid and lipid-inflammation parameters and glucose metabolism abnormalities

Subgroup analyses were performed to further explore the heterogeneity of associations for each parameter across age, sex, BMI, smoking, drinking, and hypertension groups. Overall, both individual non-traditional lipid parameters and non-traditional lipid-inflammation parameters were positively associated with glucose metabolism abnormalities across all subgroups, indicating robust findings (**Supplementary Figures 6**, **7**). Age showed significant interaction effects for all parameters (all *P* for interaction < 0.001), with stronger associations observed in individuals younger than 60 years. In addition, sex, BMI, smoking status, and hypertension exhibited significant multiplicative interactions with certain parameters in relation to glucose metabolism abnormalities.

### 3.6 Diagnostic ability of non-traditional lipid and lipid-inflammation parameters for prediabetes and diabetes

**Figure 1:**
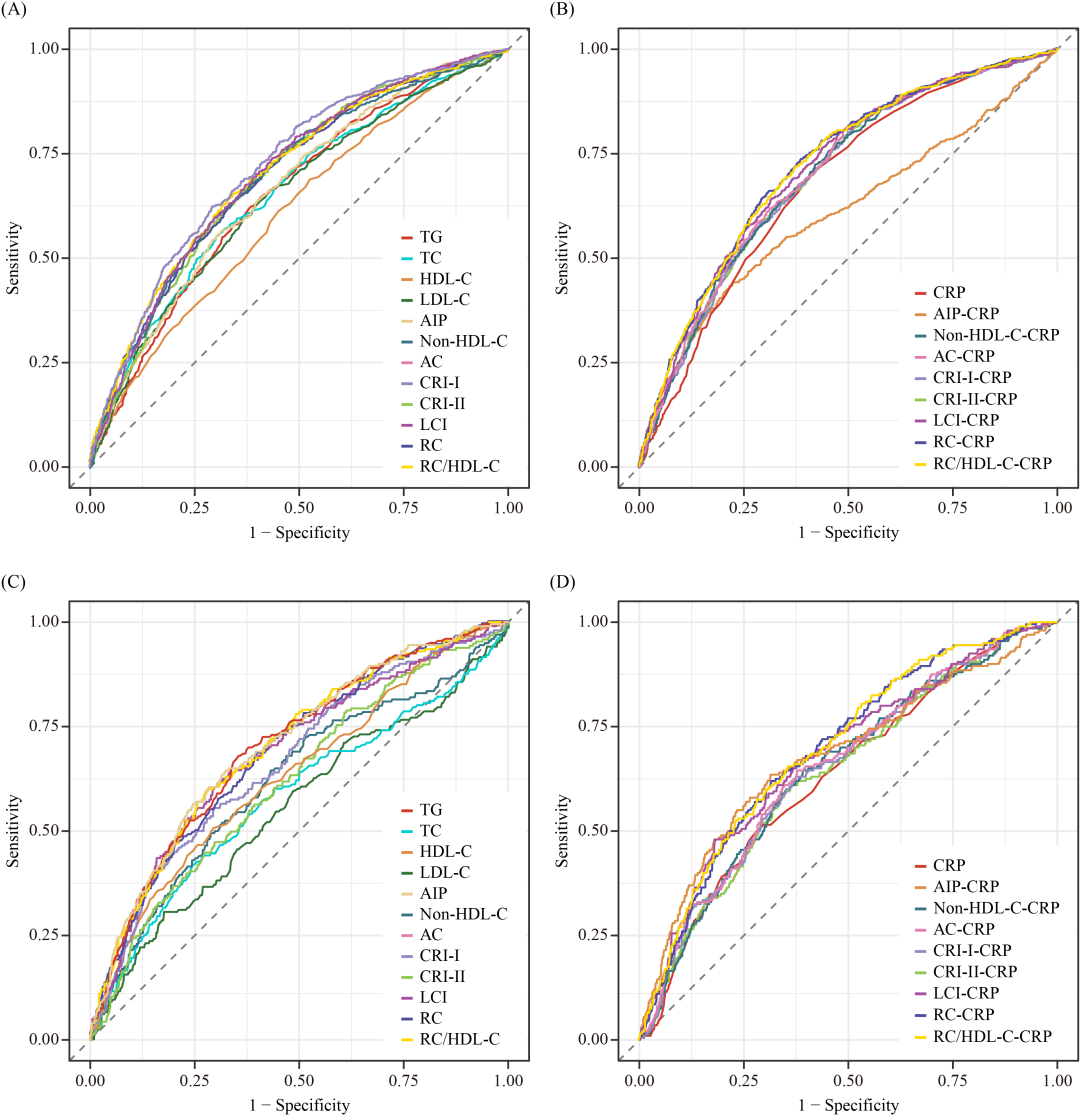
Predictive performance of lipid parameters and lipid-inflammation parameters for prediabetes and diabetes based on ROC analysis. **(A)** Lipid parameters for predicting prediabetes. **(B)** Lipid-inflammation parameters for predicting prediabetes. **(C)** Lipid parameters for predicting diabetes. **(D)** Lipid-inflammation parameters for predicting diabetes. CRP refers to high-sensitivity C-reactive protein (hs-CRP) throughout this study; TG, triglycerides; TC, total cholesterol; HDL-C, high-density lipoprotein cholesterol; LDL-C, low-density lipoprotein cholesterol; AIP, atherogenic index of plasma; LCI, lipoprotein combined Index; CRI-I, cardiovascular risk index-I; CRI-II, cardiovascular risk index-II; RC, remnant cholesterol; RC/HDL-C, remnant cholesterol/high density lipoprotein cholesterol; AC, atherogenic coefficient; Non-HDL-C, non- high density lipoprotein cholesterol.

ROC analyses were performed to evaluate the discriminatory ability of non-traditional lipid parameters and non-traditional lipid-inflammation parameters distinguishing prediabetes and diabetes (**Figure 1**, **Supplementary Tables 4**, **5**). In the prediabetes comparison, non-traditional lipid parameters generally showed higher AUC values. Among these, AC and CRI-I showing the highest performance (AUC = 0.717, 95%*CI*:0.694-0.740, *P* < 0.001; **Figure 1A**). Non-traditional lipid-inflammatory parameters also showed higher AUC values than hs-CRP, with RC-CRP and RC/HDL-C-CRP (AUC = 0.719, 95%*CI*:0.696-0.742, *P* < 0.001) showing the best performance (**Figure 1B**). In the diabetes comparison, non-traditional lipid parameters also showed slightly superior performance compared to traditional parameters, with AIP (AUC = 0.703, 95%*CI*:0.666-0.741, *P* < 0.001) demonstrating the best performance (**Figure 1C**). Among non-traditional lipid-inflammation parameters, all outperformed hs-CRP, with RC/HDL-C-CRP demonstrating the best performance (AUC = 0.694, 95%*CI*:0.657-0.731, *P* < 0.001; **Figure 1D**).

C-statistic analysis showed that adding non-traditional lipid parameters and non-traditional lipid-inflammation parameters to the baseline model resulted in modest increases in C-statistics in both the prediabetes and diabetes comparisons (*P* < 0.001). Incremental diagnostic analyses, including the NRI and IDI, demonstrated that incorporating non-traditional lipid parameters or non-traditional lipid-inflammation parameters significantly enhanced diagnostic stratification in the prediabetes comparison. In the diabetes comparison, specific non-traditional lipid parameters or non-traditional lipid-inflammation parameters also significantly improved diagnostic stratification (**Supplementary Table 6** and **S7**).

### 3.7 Mediating role of hs-CRP in the association between non-traditional lipid parameters and prediabetes and diabetes

**Figure 2:**
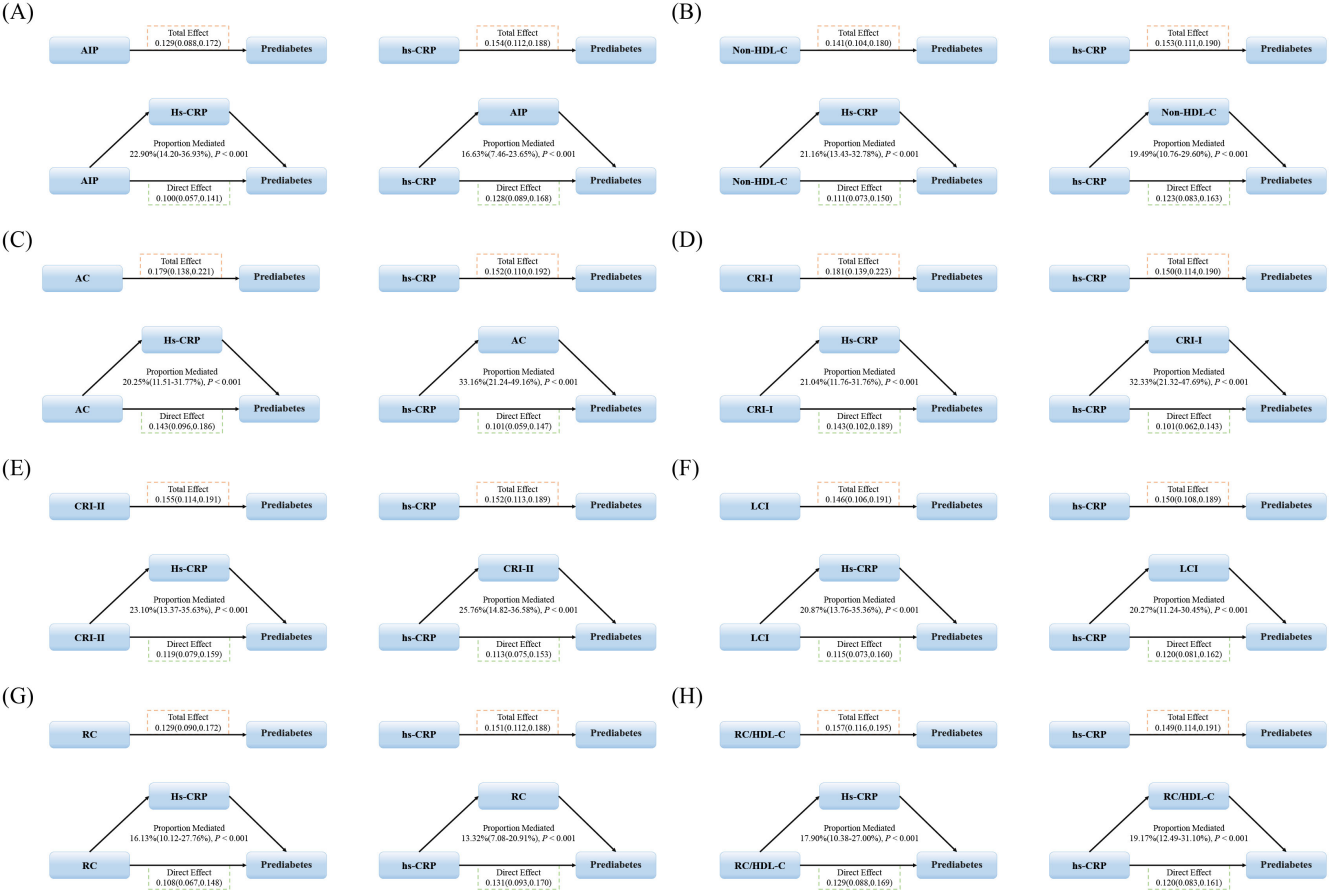
Mediation analysis of hs-CRP on the association between non-traditional lipid parameters and the risk of prediabetes. Models were adjusted for age, sex, BMI, education level, marital status, smoking, drinking status, hypertension, and dyslipidemia drug. CRP refers to high-sensitivity C-reactive protein (hs-CRP) throughout this study. **(A)** AIP, atherogenic index of plasma; **(B)** non-HDL-C, non-high-density lipoprotein cholesterol; **(C)** AC, atherogenic coefficient; **(D)** CRI-I, Castelli’s risk index I; **(E)** CRI-II, Castelli’s risk index II; **(F)** LCI, lipoprotein combine index; **(G)** RC, remnant cholesterol; and **(H)** RC/HDL-C, remnant cholesterol-to-high-density lipoprotein cholesterol ratio.

A mediation model was constructed to examine whether hs-CRP mediates the association between non-traditional lipid parameters and prediabetes and diabetes. As shown in **Figure 2** and **Supplementary Table 8**, hs-CRP partially mediated the association between eight non-traditional lipid parameters and prediabetes, with mediation proportions ranging from 16.13 to 23.10%. In addition, non-traditional lipid parameters partially mediated the association between hs-CRP and prediabetes, with mediation proportions ranging from 13.32 to 33.16% (**Figure 2**). However, as shown in **Supplementary Table 9**, no mediation effect of hs-CRP on the impact of non-traditional lipid parameters on diabetes risk was observed (mediation effect *P*>0.05).

## 4 Discussion

Our study systematically evaluated the associations of non-traditional lipid parameters and non-traditional lipid-inflammation parameters with impaired glucose metabolism in Chinese adults. Higher levels of these parameters were significantly associated with prediabetes and diabetes, with generally consistent results observed in dose-response and subgroup analyses. In the prediabetes comparison, AC, CRI-I, RC-CRP, and RC/HDL-C-CRP showed relatively higher diagnostic ability, whereas AIP and RC/HDL-C-CRP showed relatively higher diagnostic ability in the diabetes comparison. Mediation analysis revealed that hs-CRP partially mediated the association between non-traditional lipid parameters and prediabetes. Conversely, non-traditional lipid parameters also partially mediated the association between hs-CRP and prediabetes, suggesting a bidirectional association between lipid metabolism abnormalities and inflammation.

Diabetes is a complex disease, often characterized by dyslipidemia, particularly elevated triglycerides and LDL-C (26). Under pathological conditions, abnormal lipid droplet accumulation leads to ectopic lipid deposition and lipotoxicity, thereby contributing to the progression of diabetes and its complications in target organs such as the heart, kidneys, retina, and brain (27). Typically, impaired glucose metabolism is often accompanied by changes in the lipoprotein profile, including increased levels of triglyceride-rich lipoproteins, an increased proportion of small dense LDL particles, and impaired HDL-C function. These alterations may interfere with insulin signaling and reduce insulin sensitivity through lipotoxic effects (28). However, these changes mainly affect the structure, composition, and function of lipoprotein particles, rather than simply increases or decreases in cholesterol levels. Therefore, although traditional lipid parameters such as TC, TG, HDL-C, and LDL-C are widely used to monitor dyslipidemia, these indicators do not fully capture the complex lipid metabolism characteristics and the severity of IR in diabetic patients (29). In contrast to traditional lipid parameters, non-traditional lipid parameters typically comprise multiple lipid components. By incorporating features of atherogenic lipoproteins, including increased triglyceride-rich lipoproteins and small dense LDL-C as well as reduced HDL-C protective effects, these markers can more effectively characterize lipid structural abnormalities and dyslipoproteinemia through ratios or combinations (30, 31).

Our results indicate that levels of eight non-traditional lipid parameters are positively associated with glucose metabolism abnormalities to varying degrees. RCS analysis further suggests that most non-traditional lipid parameters demonstrate a nonlinear dose-response relationship with glucose metabolism abnormalities. At the same time, these indicators also demonstrate superior diagnostic ability compared with traditional lipid parameters for risk identification and improved incremental diagnostic performance. Li M et al. found that most non-traditional lipid markers were significantly associated with prediabetes in a Chinese population study, with AIP showing the strongest association (19). Another NHANES-based study also indicated that six non-traditional lipid parameters were associated with IR, and HOMA-IR mediates the adverse effects of AIP and RC on diabetes risk (32). This evidence further supports the potential value of non-traditional lipid parameters in assessing the risk of abnormal glucose metabolism, suggesting their potential as biomarkers for early identification of high-risk populations.

Inflammation is an important contributor to the development of diabetes and its complications. Persistently elevated proinflammatory cytokines such as TNF-α, IL-6, and IL-1β promote IR and disrupt glucose metabolism by impairing pancreatic β-cell function and weakening insulin action (33). Meanwhile, excess lipid accumulation may lead to ectopic lipid deposition and pro-inflammatory lipid accumulation, thereby triggering inflammatory responses in macrophages within adipose tissue and the pancreatic microenvironment, contributing to IR and diabetes (34). Our population-based study showed that non-traditional lipid-inflammation parameters were positively associated with impaired glucose metabolism. Although their effect sizes were slightly smaller than those of individual non-traditional lipid parameters, they demonstrated better diagnostic ability and incremental diagnostic performance. These findings suggest that combining lipid parameters with inflammatory markers may improve identification of high-risk individuals and provide a potential tool for early detection of impaired glucose metabolism. Mediation analysis further showed that hs-CRP partially mediated the association between non-traditional lipid parameters and prediabetes, with bidirectional mediation effects observed between hs-CRP and lipid parameters. However, no mediation effect of hs-CRP was observed for diabetes risk. This may be due to chronic inflammation promoting metabolic disorders to some extent by interfering with insulin signaling and impairing β-cell function during the early stages of glucose metabolism abnormalities (35). However, in advanced diabetes, hyperglycemia-related toxicity, along with long-term β-cell damage and other metabolic abnormalities, also contribute to the pathological process, thereby diminishing the mediating role of inflammation (36).

Subgroup analyses revealed that non-traditional lipid parameters and non-traditional lipid-inflammation parameters were positively associated with impaired glucose metabolism across all subgroups. Notably, in individuals aged <60 years, several parameters exhibited higher effect estimates for impaired glucose metabolism, and all parameters demonstrated significant multiplicative interactions with age. Chen JX et al. showed that the associations between dyslipidemia and inflammation-related biomarkers with incident T2D was strongest in younger individuals and attenuated with increasing age (37). Additionally, sex-specific differences were observed in the associations between non-traditional lipid and lipid-inflammation parameters and the risk of impaired glucose metabolism. Our study found that the associations of certain indicators were stronger in females and demonstrated a multiplicative interaction with sex, which may be partly attributable to differences in endogenous estrogen levels. Normally, estrogen confers metabolic protection by regulating lipid distribution, improving lipoprotein composition, and suppressing low-grade inflammation (38). When lipid or inflammatory levels become dysregulated, pancreatic β-cells and insulin signaling pathways in females may be more susceptible to these disturbances, thereby increasing the risk of impaired glucose metabolism (39).

Prediabetes is widely recognized as a high-risk state for the development of diabetes. Tabák et al. reported that individuals with prediabetes have an annual progression rate of 5-10% to diabetes (40). Meanwhile, numerous clinical trials have demonstrated that lifestyle interventions and medication can reduce the risk of individuals with prediabetes progressing to diabetes and may even restore normoglycemia in some individuals (41–43). Thus, since prediabetes is an intervention-responsive condition, using non-traditional lipid and lipid-inflammation parameters for early identification of individuals at higher risk, particularly with stratified monitoring and intervention targeting specific populations, holds significant importance for delaying or halting disease progression.

In this study, we evaluated the associations and diagnostic ability of eight non-traditional lipid parameters for glucose metabolism abnormalities. We identified a bidirectional mediating effect between hs-CRP and non-traditional lipid parameters in prediabetes and constructed a composite non-traditional lipid-inflammation parameters by integrating hs-CRP with these lipid parameters. This composite demonstrated superior diagnostic ability for glucose metabolism abnormalities compared with traditional indicators. However, several limitations should be noted. First, this is a cross-sectional study, and although multivariable models were used to adjust for confounders, residual confounding cannot be entirely excluded. Second, inflammatory status was assessed only by hs-CRP, which may not fully capture the complexity of inflammatory pathways. Third, non-traditional lipid parameters are derived from conventional lipid measurements and provide integrated information on lipid metabolism. Fourth, given the cross-sectional design of this study, the observed mediating effects should be interpreted cautiously as exploratory and statistical in nature. Fifth, although incremental diagnostic performance was assessed using C-statistics, NRI, and IDI, all analyses were conducted within the same dataset without external validation or calibration. In addition, the improvements in some C-statistics were relatively small, and their clinical significance should therefore be interpreted with caution. Finally, although we explored the mediating role of inflammation and assessed the predictive value of each parameter pair, longitudinal follow-up and experimental validation are lacking. Prospective studies and mechanistic research are needed to confirm these findings.

## 5 Conclusion

Non-traditional lipid parameters and non-traditional lipid-inflammation parameters are associated with impaired glucose metabolism, and the observed associations involving inflammation suggest a potential mediating role of inflammatory pathways in the relationship between lipid abnormalities and glucose dysregulation. Integrating inflammatory markers into lipid assessments may enhance diagnostic ability of high-risk individuals, particularly those with prediabetes, providing robust epidemiological evidence to support early detection and prevention strategies.

## Data Availability

The raw data supporting the conclusions of this article will be made available by the authors, without undue reservation.
