# Supplementary material for: Nontraditional lipid and lipid-inflammatory parameters for risk stratification of abnormal glucose metabolism: a cross-sectional study in Chinese adults
Source: Front Endocrinol (Lausanne). 2026 Jul 10;17:1873940. doi: 10.3389/fendo.2026.1873940 (PMC13395609; doi:10.3389/fendo.2026.1873940)
Supplement: Supplementary file 1 [file DataSheet1.docx]

Supplementary Method 1 Data collection

A standardized structured questionnaire was administered by interviewers who had undergone uniform training and passed assessments, through face-to-face interviews with respondents. The questionnaire included sociodemographic characteristics (age, sex, ethnicity, occupation, educational level, marital status, etc.), lifestyle factors (smoking, alcohol consumption, dietary patterns, physical activity, etc.), and medical history (including hypertension, dyslipidemia, diabetes, coronary heart disease, and stroke, etc.). The physical examination included measurements of height, weight, body fat percentage, waist circumference, blood pressure, and other parameters. Blood parameter testing was conducted by professional laboratory technicians. After blood sample collection, complete blood counts were performed at the monitoring site laboratory. To ensure nationwide comparability of samples, biochemical parameters were analyzed by a third-party independent medical laboratory accredited under the ISO 15189 quality management system for medical laboratories and without foreign investment. Among these, glycated hemoglobin (HbA1c) was measured using high-performance liquid chromatography; fasting blood glucose (FBG), triglycerides (TG), and total cholesterol (TC) were determined by the oxidase method; and high-density lipoprotein cholesterol (HDL-C) and low-density lipoprotein cholesterol (LDL-C) were measured using a one-step method.

Supplementary Method 2 Calculation of non-traditional lipid and non-traditional lipid-inflammation parameters

Non-Traditional Lipid Parameters

(1) Atherogenic index of plasma (AIP) = lg (TG/HDL-C);

(2) Non-HDL-C = TC-HDL-C;

(3) Atherogenic coefficient (AC) = Non-HDL-C/HDL-C;

(4) Castelli’s index-I (CRI-I) = TC/HDL-C;

(5) Castelli’s index-II (CRI-II) = LDL-C/HDL-C;

(6) Lipoprotein combine index (LCI) = TC×TG×LDL-C/HDL-C;

(7) Remnant cholesterol (RC) = TC-HDL-C-LDL-C;

(8) RC/HDL-C ratio = RC/HDL-C;

Non-Traditional Lipid-Inflammation Parameters

(1) AIP-CRP = lg (TG/HDL-C) × hs-CRP /10;

(2) Non-HDL-C-CRP = TC−HDL-C × hs-CRP /10;

(3) AC-CRP = Non-HDL-C/HDL-C × hs-CRP /10;

(4) CRI-I-CRP = TC/HDL-C × hs-CRP /10;

(5) CRI-II-CRP = LDL-C/HDL-C × hs-CRP /10;

(6) LCI-CRP = TC×TG×LDL-C/HDL-C × hs-CRP /10;

(7) RC-CRP = TC-HDL-C-LDL-C × hs-CRP /10;

(8) RC/HDL-C-CRP = RC/HDL-C × hs-CRP /10;

Supplementary Method 3 Assessment of multicollinearity and correlations among covariates

Prior to multiple regression analysis, multicollinearity among covariates was assessed by calculating the variance inflation factor (VIF) using the collinear R package. Correlations among covariates were evaluated using the Spearman correlation coefficient for continuous variables, Cramér’s V coefficient for categorical variables, and the point-biserial correlation coefficient for continuous variables paired with binary variables.

| Table S1 Comparison of baseline characteristics between included and excluded participants | | | | |
| --- | --- | --- | --- | --- |
| Variables | Overall | Pre-Diabetes | Diabetes | *P*-Value |
|  | （n=9,790） | （n=7,489） | （n=2,301） |  |
| Age (years) | 43 (30, 57) | 53 (44, 67) | 43 (30, 58) | 0.048 |
| Sex, n (%) |  |  |  | 0.942 |
| Male | 4,810 (49.13%) | 3,681 (49.15%) | 1,129 (49.07%) |  |
| Female | 4,980 (50.87%) | 3,808 (50.85%) | 1,172 (5.93%) |  |
| BMI (kg/m^2^) | 23.4 (21.1, 26.0) | 23.3 (21.1, 25.9) | 23.8 (21.3, 26.4) | <0.001 |
| Educational level, n (%) |  |  |  | 0.919 |
| Less than high school | 5,204 (53.16%) | 3,983 (53.18%) | 1,221 (53.06%) |  |
| High school and above | 4,586 (46.84%) | 3,506 (46.82%) | 1,080 (46.94%) |  |
| Marital status, n (%) |  |  |  | 0.699 |
| Married/Living with partner | 7,047 (71.98%) | 5,398 (72.08%) | 652 (28.34%) |  |
| Others | 2,743 (28.02%) | 2,091 (27.92%) | 1,649 (71.66%) |  |
| Smoking status, n (%) |  |  |  | 0.574 |
| Yes | 2,229 (22.77%) | 1,715 (22.90%) | 514 (22.34%) |  |
| No | 7,561 (77.23%) | 5,774 (77.10%) | 1,787 (77.66%) |  |
| Drinking status, n (%) |  |  |  | 0.054 |
| Yes | 3,171 (32.39%) | 2,466 (32.93%) | 705 (30.64%) |  |
| No | 6,619 (67.61%) | 5,023 (67.07%) | 1,596 (69.36%) |  |
| Hypertension, n (%) |  |  |  | <0.001 |
| Yes | 3,330 (34.01%) | 2,625 (35.05%) | 705 (30.64%) |  |
| No | 6,460 (65.99%) | 4,864 (64.95%) | 1,596 (69.36%) |  |
| Dyslipidemia drug, n (%) |  |  |  | 0.092 |
| Yes | 108 (1.10%) | 90 (1.20%) | 18 (0.78%) |  |
| No | 9,682 (98.90%) | 7,399 (98.80%) | 2,283 (99.22%) |  |

| Table S2 Quartile cut-off values of all non-traditional lipid and lipid-inflammatory parameters | | | | |
| --- | --- | --- | --- | --- |
| Variable | Q1 | Q2 | Q3 | Q4 |
| AIP | ≤ -0.313 | -0.313, -0.117 | -0.117, 0.079 | >0.079 |
| NON_HDL | ≤ 51.66 | 51.66, 62.64 | 62.64, 74.34 | >74.34 |
| AC | ≤ 1.905 | 1.905, 2.438 | 2.438, 3.08 | >3.08 |
| CRI_I | ≤ 2.905 | 2.905, 3.438 | 3.438, 4.08 | >4.08 |
| CRI_II | ≤ 1.72 | 1.72, 2.173 | 2.173, 2.689 | >2.689 |
| LCI | ≤ 2027.876 | 2027.876, 3671.441 | 3671.441, 7057.44 | >7057.44 |
| RC | ≤ 3.6 | 3.6, 6.3 | 6.3, 9.72 | >9.72 |
| RC_HDL | ≤ 0.131 | 0.131, 0.241 | 0.241, 0.405 | >0.405 |
| AIP_CRP | ≤ -0.032 | -0.032, -0.011 | -0.011, 0.015 | >0.015 |
| NON_HDL_CRP | ≤ 4.52 | 4.52, 9.612 | 9.612, 19.467 | >19.467 |
| AC_CRP | ≤ 0.166 | 0.166, 0.376 | 0.376, 0.819 | >0.819 |
| CRI_I_CRP | ≤ 0.247 | 0.247, 0.535 | 0.535, 1.108 | >1.108 |
| CRI_II_CRP | ≤ 0.149 | 0.149, 0.333 | 0.333, 0.708 | >0.708 |
| LCI_CRP | ≤ 194.394 | 194.394, 587.569 | 587.569, 1832.17 | >1832.17 |
| RC_CRP | ≤ 0.302 | 0.302, 0.891 | 0.891, 2.318 | >2.318 |
| RC_HDL_CRP | ≤ 0.011 | 0.011, 0.035 | 0.035, 0.098 | >0.098 |
| RC_HDL_CRP | ≤ 0.011 | 0.011, 0.035 | 0.035, 0.098 | >0.098 |
| CRP refers to high-sensitivity C-reactive protein (hs-CRP) throughout this study; TG, triglycerides; TC, total cholesterol; HDL-C, high-density lipoprotein cholesterol; LDL-C, low-density lipoprotein cholesterol; AIP, atherogenic index of plasma; LCI, lipoprotein combined Index; CRI-I, cardiovascular risk index-I; CRI-II, cardiovascular risk index-II; RC, remnant cholesterol; RC/HDL-C, remnant cholesterol/high density lipoprotein cholesterol; AC, atherogenic coefficient; Non-HDL-C, non- high density lipoprotein cholesterol. | | | | |

| Table S3 Distribution characteristics of participants' blood parameters | | | | | |
| --- | --- | --- | --- | --- | --- |
| Variables | Overall | Non-Diabetes | Pre-Diabetes | Diabetes | *P*-Value |
|  | （n=2301） | （n=1393） | （n=708） | （n=200） |  |
| TG | 19 (13, 28) | 17 (12, 24) | 23 (16, 33) | 29 (19, 43) | <0.001 |
| TC | 88 (78, 101) | 85 (76, 96) | 96 (84, 107) | 95 (79, 109) | <0.001 |
| HDL-C | 25.6 (22.3, 29.2) | 26.5 (23.2, 30.1) | 24.7 (21.4, 27.7) | 23.0 (20.0, 27.7) | <0.001 |
| LDL-C | 55 (46, 66) | 52 (44, 62) | 60 (50, 71) | 58 (47, 70) | <0.001 |
| CRP | 1.50 (0.80, 2.80) | 1.20 (0.70, 2.20) | 2.10 (1.30, 3.40) | 2.40 (1.20, 4.10) | <0.001 |
| AIP | -0.12 (-0.31, 0.08) | -0.19 (-0.37, -0.01) | -0.03 (-0.21, 0.16) | 0.09 (-0.13, 0.32) | <0.001 |
| Non-HDL-C | 63 (52, 74) | 57 (49, 69) | 70 (59, 82) | 71 (59, 84) | <0.001 |
| AC | 2.44 (1.90, 3.08) | 2.18 (1.74, 2.76) | 2.87 (2.32, 3.42) | 2.98 (2.33, 3.69) | <0.001 |
| CRI_I | 3.44 (2.90, 4.08) | 3.18 (2.74, 3.76) | 3.87 (3.32, 4.42) | 3.98 (3.33, 4.69) | <0.001 |
| CRI_II | 2.17 (1.72, 2.69) | 1.97 (1.59, 2.47) | 2.49 (2.03, 2.92) | 2.45 (1.98, 3.09) | <0.001 |
| LCI | 3,671 (2,028, 7,057) | 2,806 (1,648, 5,012) | 5,357 (3,134, 9,335) | 7,357 (3,600, 12,268) | <0.001 |
| RC | 6.3 (3.6, 9.7) | 5.0 (2.7, 7.9) | 8.3 (5.4, 12.4) | 9.3 (6.2, 14.9) | <0.001 |
| RC/HDL-C | 0.24 (0.13, 0.41) | 0.19 (0.10, 0.31) | 0.33 (0.20, 0.52) | 0.41 (0.24, 0.70) | <0.001 |
| AIP-CRP | -0.01 (-0.03, 0.01) | -0.02 (-0.03, 0.00) | 0.00 (-0.03, 0.04) | 0.02 (-0.02, 0.10) | <0.001 |
| Non-HDL-C-CRP | 10 (5, 19) | 7 (3, 14) | 15 (8, 26) | 16 (8, 30) | <0.001 |
| AC-CRP | 0.38 (0.17, 0.82) | 0.27 (0.12, 0.57) | 0.62 (0.31, 1.12) | 0.69 (0.30, 1.41) | <0.001 |
| CRI-I-CRP | 0.53 (0.25, 1.11) | 0.40 (0.19, 0.79) | 0.84 (0.45, 1.45) | 0.94 (0.44, 1.86) | <0.001 |
| CRI-II-CRP | 0.33 (0.15, 0.71) | 0.25 (0.11, 0.51) | 0.53 (0.27, 0.96) | 0.60 (0.25, 1.15) | <0.001 |
| LCI-CRP | 588 (194, 1,832) | 350 (119, 996) | 1,227 (467, 3,020) | 1,704 (513, 4,631) | <0.001 |
| RC-CRP | 0.89 (0.30, 2.32) | 0.55 (0.19, 1.40) | 1.66 (0.75, 3.85) | 2.34 (0.86, 4.81) | <0.001 |
| RC/HDL-C-CRP | 0.03 (0.01, 0.10) | 0.02 (0.01, 0.06) | 0.07 (0.03, 0.16) | 0.10 (0.03, 0.24) | <0.001 |
| CRP refers to high-sensitivity C-reactive protein (hs-CRP) throughout this study; TG, triglycerides; TC, total cholesterol; HDL-C, high-density lipoprotein cholesterol; LDL-C, low-density lipoprotein cholesterol; AIP, atherogenic index of plasma; LCI, lipoprotein combined Index; CRI-I, cardiovascular risk index-I; CRI-II, cardiovascular risk index-II; RC, remnant cholesterol; RC/HDL-C, remnant cholesterol/high density lipoprotein cholesterol; AC, atherogenic coefficient; Non-HDL-C, non- high density lipoprotein cholesterol. | | | | | |

| Table S4 The AUC, best threshold, sensitivity, and specificity of lipid parameters in prediabetes | | | | | |
| --- | --- | --- | --- | --- | --- |
| Variables | AUC (95% *CI*) | Best threshold | Specificity | Sensitivity | *P* value |
| Traditional lipid parameters | | | | | |
| TG | 0.656(0.631,0.680) | 19.890 | 0.620 | 0.620 | < 0.001 |
| TC | 0.656(0.631,0.681) | 93.330 | 0.697 | 0.551 | < 0.001 |
| HDL-C | 0.609(0.583,0.634) | 26.730 | 0.475 | 0.688 | < 0.001 |
| LDL-C | 0.647(0.622,0.672) | 54.630 | 0.576 | 0.658 | < 0.001 |
| Non-traditional lipid parameters | | | | | |
| AIP | 0.663(0.639,0.687) | -0.057 | 0.693 | 0.556 | < 0.001 |
| Non-HDL-C | 0.697(0.673,0.720) | 66.150 | 0.698 | 0.603 | < 0.001 |
| AC | 0.717(0.694,0.740) | 2.619 | 0.707 | 0.623 | < 0.001 |
| CRI-I | 0.717(0.694,0.740) | 3.619 | 0.707 | 0.623 | < 0.001 |
| CRI-II | 0.691(0.667,0.714) | 2.164 | 0.607 | 0.688 | < 0.001 |
| LCI | 0.702(0.678,0.725) | 3244.986 | 0.565 | 0.739 | < 0.001 |
| RC | 0.694(0.670,0.717) | 5.760 | 0.579 | 0.713 | < 0.001 |
| RC/HDL-C | 0.701(0.678,0.725) | 0.252 | 0.656 | 0.654 | < 0.001 |
| Non-traditional lipid- inflammatory parameter**s** | | | | | |
| hs-CRP | 0.678(0.654,0.701) | 1.450 | 0.589 | 0.702 | < 0.001 |
| AIP-CRP | 0.606(0.579,0.633) | 0.002 | 0.776 | 0.441 | < 0.001 |
| Non-HDL-C -CRP | 0.698(0.675,0.722) | 8.179 | 0.546 | 0.756 | < 0.001 |
| AC-CRP | 0.702(0.679,0.725) | 0.276 | 0.511 | 0.805 | < 0.001 |
| CRI-I-CRP | 0.697(0.673,0.720) | 0.397 | 0.500 | 0.802 | < 0.001 |
| CRI-II-CRP | 0.695(0.672,0.718) | 0.259 | 0.520 | 0.781 | < 0.001 |
| LCI-CRP | 0.714(0.691,0.737) | 443.752 | 0.561 | 0.766 | < 0.001 |
| RC-CRP | 0.719(0.696,0.742) | 0.806 | 0.612 | 0.734 | < 0.001 |
| RC/HDL-C-CRP | 0.719(0.696,0.742) | 0.033 | 0.627 | 0.718 | < 0.001 |
| CRP refers to high-sensitivity C-reactive protein (hs-CRP) throughout this study; TG, triglycerides; TC, total cholesterol; HDL-C, high-density lipoprotein cholesterol; LDL-C, low-density lipoprotein cholesterol; AIP, atherogenic index of plasma; LCI, lipoprotein combined Index; CRI-I, cardiovascular risk index-I; CRI-II, cardiovascular risk index-II; RC, remnant cholesterol; RC/HDL-C, remnant cholesterol/high density lipoprotein cholesterol; AC, atherogenic coefficient; Non-HDL-C, non- high density lipoprotein cholesterol. | | | | | |

| Table S5 The AUC, best threshold, sensitivity, and specificity of lipid parameters in diabetes | | | | | |
| --- | --- | --- | --- | --- | --- |
| Variables | AUC (95% *CI*) | Best threshold | Specificity | Sensitivity | *P* value |
| Traditional lipid parameters | | | | | |
| TG | 0.700(0.662,0.738) | 23.130 | 0.644 | 0.680 | < 0.001 |
| TC | 0.579(0.533,0.625) | 99.630 | 0.747 | 0.425 | < 0.001 |
| HDL-C | 0.632(0.590,0.673) | 23.130 | 0.707 | 0.505 | < 0.001 |
| LDL-C | 0.554(0.509,0.599) | 68.850 | 0.825 | 0.305 | 0.020 |
| Non-traditional lipid parameters | | | | | |
| AIP | 0.703(0.666,0.741) | 0.025 | 0.716 | 0.605 | < 0.001 |
| Non-HDL-C | 0.620(0.576,0.663) | 64.710 | 0.570 | 0.645 | < 0.001 |
| AC | 0.664(0.624,0.704) | 2.885 | 0.705 | 0.555 | < 0.001 |
| CRI-I | 0.664(0.624,0.704) | 3.885 | 0.705 | 0.555 | < 0.001 |
| CRI-II | 0.621(0.580,0.662) | 2.560 | 0.704 | 0.475 | < 0.001 |
| LCI | 0.685(0.645,0.725) | 5538.575 | 0.696 | 0.615 | < 0.001 |
| RC | 0.687(0.649,0.725) | 7.200 | 0.600 | 0.685 | < 0.001 |
| RC/HDL-C | 0.699(0.662,0.737) | 0.356 | 0.720 | 0.600 | < 0.001 |
| Non-traditional lipid- inflammatory parameter**s** | | | | | |
| hs-CRP | 0.634(0.594,0.674) | 2.350 | 0.703 | 0.515 | < 0.001 |
| AIP-CRP | 0.678(0.635,0.720) | 0.000 | 0.686 | 0.635 | < 0.001 |
| Non-HDL-C -CRP | 0.646(0.607,0.685) | 12.303 | 0.609 | 0.645 | < 0.001 |
| AC-CRP | 0.653(0.614,0.692) | 0.506 | 0.621 | 0.645 | < 0.001 |
| CRI-I-CRP | 0.650(0.610,0.689) | 0.704 | 0.626 | 0.630 | < 0.001 |
| CRI-II-CRP | 0.639(0.599,0.679) | 0.496 | 0.653 | 0.595 | < 0.001 |
| LCI-CRP | 0.681(0.641,0.720) | 2292.774 | 0.819 | 0.480 | < 0.001 |
| RC-CRP | 0.694(0.657,0.731) | 1.640 | 0.684 | 0.620 | < 0.001 |
| RC/HDL-C-CRP | 0.697(0.660,0.734) | 0.056 | 0.644 | 0.655 | < 0.001 |
| CRP refers to high-sensitivity C-reactive protein (hs-CRP) throughout this study; TG, triglycerides; TC, total cholesterol; HDL-C, high-density lipoprotein cholesterol; LDL-C, low-density lipoprotein cholesterol; AIP, atherogenic index of plasma; LCI, lipoprotein combined Index; CRI-I, cardiovascular risk index-I; CRI-II, cardiovascular risk index-II; RC, remnant cholesterol; RC/HDL-C, remnant cholesterol/high density lipoprotein cholesterol; AC, atherogenic coefficient; Non-HDL-C, non- high density lipoprotein cholesterol. | | | | | |

| Table S6 Incremental diagnostic ability of non-traditional lipid and lipid-inflammatory parameters in prediabetes | | | | | | |
| --- | --- | --- | --- | --- | --- | --- |
|  | **C Statistics** | | **NRI** | | **IDI** | |
|  | **Estimate (95%CI)** | ***P* value** | **Estimate (95%CI)** | ***P* value** | **Estimate (95%CI)** | ***P* value** |
| Basic model | 0.780 (0.760,0.801) | <0.001 | Ref. |  | Ref. |  |
| Basic model + CRP | 0.795 (0.776,0.815) | <0.001 | 0.071 (0.038,0.106) | <0.001 | 0.026 (0.019,0.033) | <0.001 |
| Basic model + AIP | 0.789 (0.769,0.809) | <0.001 | 0.036 (0.006,0.063) | 0.013 | 0.018 (0.012,0.024) | <0.001 |
| Basic model + AIP-CRP | 0.789 (0.769,0.809) | <0.001 | 0.046 (0.018,0.074) | 0.001 | 0.019 (0.013,0.026) | <0.001 |
| Basic model + Non-HDL-C | 0.794 (0.774,0.814) | <0.001 | 0.068 (0.038,0.098) | <0.001 | 0.024 (0.017,0.031) | <0.001 |
| Basic model + Non-HDL-C-CRP | 0.795 (0.775,0.815) | <0.001 | 0.080 (0.045,0.115) | <0.001 | 0.025 (0.018,0.031) | <0.001 |
| Basic model + AC | 0.800 (0.781,0.820) | <0.001 | 0.101(0.071,0.134) | <0.001 | 0.036 (0.027,0.044) | <0.001 |
| Basic model + AC-CRP | 0.798 (0.779,0.817) | <0.001 | 0.109 (0.072,0.146) | <0.001 | 0.030 (0.023,0.038) | <0.001 |
| Basic model + CRI-I | 0.800 (0.781,0.820) | <0.001 | 0.101 (0.071,0.134) | <0.001 | 0.036 (0.027,0.044) | <0.001 |
| Basic model + CRI-I-CRP | 0.796 (0.776,0.816) | <0.001 | 0.087 (0.050,0.122) | <0.001 | 0.027 (0.020,0.034) | <0.001 |
| Basic model + CRI-II | 0.796 (0.776,0.816) | <0.001 | 0.082 (0.047,0.117) | <0.001 | 0.027 (0.020,0.034) | <0.001 |
| Basic model + CRI-II-CRP | 0.796 (0.776,0.816) | <0.001 | 0.084 (0.047,0.118) | <0.001 | 0.026 (0.019,0.033) | <0.001 |
| Basic model + LCI | 0.793(0.773,0.812) | <0.001 | 0.092 (0.058,0.123) | <0.001 | 0.022 (0.016,0.029) | <0.001 |
| Basic model + LCI-CRP | 0.796 (0.776,0.816) | <0.001 | 0.100 (0.066,0.133) | <0.001 | 0.028 (0.020,0.035) | <0.001 |
| Basic model + RC | 0.791 (0.771,0.810) | <0.001 | 0.081 (0.047,0.114) | <0.001 | 0.018 (0.011,0.024) | <0.001 |
| Basic model + RC-CRP | 0.798 (0.778,0.817) | <0.001 | 0.118 (0.084,0.156) | <0.001 | 0.030 (0.023,0.038) | <0.001 |
| Basic model + RC/HDL-C | 0.797 (0.778,0.816) | <0.001 | 0.104 (0.069,0.142) | <0.001 | 0.027 (0.020,0.035) | <0.001 |
| Basic model + RC/HDL-C-CRP | 0.798 (0.779,0.817) | <0.001 | 0.106 (0.071,0.140) | <0.001 | 0.032 (0.024,0.039) | <0.001 |
| The basic model included variables from the core model, including age, sex, BMI, education level, marital status, smoking, drinking status, hypertension, and dyslipidemia drug. All lipid parameters were divided into two groups based on optimal cutoff values. CRP refers to high-sensitivity C-reactive protein (hs-CRP) throughout this study; TG, triglycerides; TC, total cholesterol; HDL-C, high-density lipoprotein cholesterol; LDL-C, low-density lipoprotein cholesterol; AIP, atherogenic index of plasma; LCI, lipoprotein combined Index; CRI-I, cardiovascular risk index-I; CRI-II, cardiovascular risk index-II; RC, remnant cholesterol; RC/HDL-C, remnant cholesterol/high density lipoprotein cholesterol; AC, atherogenic coefficient; Non-HDL-C, non- high density lipoprotein cholesterol. | | | | | | |

| Table S7 Incremental diagnostic ability of non-traditional lipid and lipid-inflammatory parameters in diabetes | | | | | | |
| --- | --- | --- | --- | --- | --- | --- |
|  | **C Statistics** | | **NRI** | | **IDI** | |
|  | **Estimate (95%CI)** | ***P* value** | **Estimate (95%CI)** | ***P* value** | **Estimate (95%CI)** | ***P* value** |
| Basic model | 0.805 (0.778,0.833) | <0.001 | Ref. |  | Ref. |  |
| Basic model + CRP | 0.807 (0.780,0.834) | <0.001 | 0.057 (0.008,0.106) | 0.029 | 0.003(0.000,0.007) | 0.048 |
| Basic model + AIP | 0.823 (0.796,0.850) | <0.001 | 0.156 (0.070,0.242) | <0.001 | 0.025 (0.014,0.035) | <0.001 |
| Basic model + AIP-CRP | 0.822 (0.795,0.849) | <0.001 | 0.145 (0.053,0.232) | 0.001 | 0.022 (0.013,0.032) | <0.001 |
| Basic model + Non-HDL-C | 0.807 (0.780,0.834) | <0.001 | 0.015 (-0.048,0.075) | 0.629 | 0.002 (-0.002,0.005) | 0.327 |
| Basic model + Non-HDL-C-CRP | 0.809 (0.781,0.836) | <0.001 | 0.056 (-0.008,0.116) | 0.087 | 0.004 (0.000,0.009) | 0.056 |
| Basic model + AC | 0.812 (0.785,0.839) | <0.001 | 0.126 (0.046,0.200) | 0.001 | 0.007 (0.000,0.014) | 0.026 |
| Basic model + AC-CRP | 0.809 (0.782,0.837) | <0.001 | 0.076 (0.008,0.139) | 0.022 | 0.006 (0.001,0.010) | 0.019 |
| Basic model + CRI-I | 0.812 (0.785,0.839) | <0.001 | 0.126(0.046,0.200) | 0.001 | 0.007 (0.000,0.014) | 0.026 |
| Basic model + CRI-I-CRP | 0.808 (0.781,0.836) | <0.001 | 0.043 (-0.019,0.101) | 0.167 | 0.005 (0.001,0.009) | 0.021 |
| Basic model + CRI-II | 0.808 (0.780,0.835) | <0.001 | 0.071 (0.015,0.127) | 0.015 | 0.002 (-0.001,0.006) | 0.174 |
| Basic model + CRI-II-CRP | 0.808 (0.781,0.836) | <0.001 | 0.042 (-0.022,0.102) | 0.186 | 0.005 (0.001,0.009) | 0.032 |
| Basic model + LCI | 0.817 (0.791,0.843) | <0.001 | 0.132 (0.051,0.208) | 0.001 | 0.013 (0.004,0.021) | 0.002 |
| Basic model + LCI-CRP | 0.818 (0.792,0.845) | <0.001 | 0.115 (0.035,0.197) | 0.005 | 0.015 (0.006,0.024) | 0.001 |
| Basic model + RC | 0.811 (0.783,0.838) | <0.001 | 0.056 (-0.018,0.130) | 0.145 | 0.009 (0.004,0.015) | 0.002 |
| Basic model + RC-CRP | 0.811 (0.784,0.838) | <0.001 | 0.099(0.016,0.180) | 0.014 | 0.009 (0.003,0.015) | 0.003 |
| Basic model + RC/HDL-C | 0.817 (0.790,0.844) | <0.001 | 0.122(0.029,0.208) | 0.006 | 0.018 (0.009,0.026) | <0.001 |
| Basic model + RC/HDL-C-CRP | 0.809 (0.782,0.836) | <0.001 | 0.073 (0.000,0.141) | 0.040 | 0.006 (0.001,0.011) | 0.014 |
| The basic model included variables from the core model, including age, sex, BMI, education level, marital status, smoking, drinking status, hypertension, and dyslipidemia drug. All lipid parameters were divided into two groups based on optimal cutoff values. CRP refers to high-sensitivity C-reactive protein (hs-CRP) throughout this study; TG, triglycerides; TC, total cholesterol; HDL-C, high-density lipoprotein cholesterol; LDL-C, low-density lipoprotein cholesterol; AIP, atherogenic index of plasma; LCI, lipoprotein combined Index; CRI-I, cardiovascular risk index-I; CRI-II, cardiovascular risk index-II; RC, remnant cholesterol; RC/HDL-C, remnant cholesterol/high density lipoprotein cholesterol; AC, atherogenic coefficient; Non-HDL-C, non- high density lipoprotein cholesterol. | | | | | | |

| Table S8 Mediation analysis of hs-CRP on the association between non-traditional lipid parameters and the risk of prediabetes | | |
| --- | --- | --- |
|  | Using hs-CRP as a mediator | Using non-traditional lipid parameters as a mediator |
| AIP |  |  |
| Total Effect | 0.129(0.088,0.172) | 0.154(0.112,0.188) |
| Direct Effect | 0.100(0.057,0.141) | 0.128(0.089,0.168) |
| Mediated Proportion (%) | 22.90(14.20,36.93) | 16.63(7.46,23.65) |
| Mediated Effect *P*-value | <0.001 | <0.001 |
| Non-HDL-C |  |  |
| Total Effect | 0.141(0.104,0.180) | 0.153(0.111,0.190) |
| Direct Effect | 0.111(0.073,0.150) | 0.123(0.083,0.163) |
| Mediated Proportion (%) | 21.16(13.43,32.78) | 19.49(10.76,29.60) |
| Mediated Effect *P*-value | <0.001 | <0.001 |
| AC |  |  |
| Total Effect | 0.179(0.138,0.221) | 0.152(0.110,0.192) |
| Direct Effect | 0.143(0.096,0.186) | 0.101(0.059,0.147) |
| Mediated Proportion (%) | 20.25(11.51,31.77) | 33.16(21.24,49.16) |
| Mediated Effect *P*-value | <0.001 | <0.001 |
| CRI-I |  |  |
| Total Effect | 0.181(0.139,0.223) | 0.150(0.114,0.190) |
| Direct Effect | 0.143(0.102,0.189) | 0.101(0.062,0.143) |
| Mediated Proportion (%) | 21.04(11.76,31.76) | 32.33(21.32,47.69) |
| Mediated Effect *P*-value | <0.001 | <0.001 |
| CRI-II |  |  |
| Total Effect | 0.155(0.114,0.191) | 0.152(0.113,0.189) |
| Direct Effect | 0.119(0.079,0.159) | 0.113(0.075,0.153) |
| Mediated Proportion (%) | 23.10(13.37,35.63) | 25.76(14.82,36.58) |
| Mediated Effect *P*-value | <0.001 | <0.001 |
| LCI |  |  |
| Total Effect | 0.146(0.106,0.191) | 0.150(0.108,0.189) |
| Direct Effect | 0.115(0.073,0.160) | 0.120(0.081,0.162) |
| Mediated Proportion (%) | 20.87(13.76,35.36) | 20.27(11.24,30.45) |
| Mediated Effect *P*-value | <0.001 | <0.001 |
| RC |  |  |
| Total Effect | 0.129(0.090,0.172) | 0.151(0.112,0.188) |
| Direct Effect | 0.108(0.067,0.148) | 0.131(0.093,0.170) |
| Mediated Proportion (%) | 16.13(10.12,27.76) | 13.32(7.08,20.91) |
| Mediated Effect *P*-value | <0.001 | <0.001 |
| RC/HDL-C |  |  |
| Total Effect | 0.157(0.116,0.195) | 0.149(0.114,0.191) |
| Direct Effect | 0.129(0.088,0.169) | 0.120(0.083,0.161) |
| Mediated Proportion (%) | 17.90(10.38,27.00) | 19.17(12.49,31.10) |
| Mediated Effect *P*-value | <0.001 | <0.001 |
| Models were adjusted for age, sex, BMI, education level, marital status, smoking, drinking status, hypertension, and dyslipidemia drug. AIP, atherogenic index of plasma; LCI, lipoprotein combined Index; CRI-I, cardiovascular risk index-I; CRI-II, cardiovascular risk index-II; RC, remnant cholesterol; RC/HDL-C, remnant cholesterol/high density lipoprotein cholesterol; AC, atherogenic coefficient; Non-HDL-C, non- high density lipoprotein cholesterol. | | |

| Table S9 Mediation analysis of hs-CRP on the association between non-traditional lipid parameters and the risk of diabetes | | |
| --- | --- | --- |
|  | Using hs-CRP as a mediator | Using non-traditional lipid parameters as a mediator |
| AIP |  |  |
| Total Effect | 0.076(0.051,0.101) | 0.023(-0.001,0.049) |
| Direct Effect | 0.074(0.049,0.100) | 0.008(-0.016,0.032) |
| Mediated Proportion (%) | NA | NA |
| Mediated Effect *P*-value | 0.510 | 0.060 |
| Non-HDL-C |  |  |
| Total Effect | 0.023(-0.001,0.047) | 0.023(0.001,0.045) |
| Direct Effect | 0.020(-0.006,0.044) | 0.019(-0.003,0.042) |
| Mediated Proportion (%) | NA | NA |
| Mediated Effect *P*-value | 0.170 | 0.138 |
| AC |  |  |
| Total Effect | 0.043(0.019,0.065) | 0.023(0.003,0.047) |
| Direct Effect | 0.039(0.015,0.063) | 0.011(-0.010,0.035) |
| Mediated Proportion (%) | NA | 49.11(13.69,288.78) |
| Mediated Effect *P*-value | 0.354 | 0.030 |
| CRI-I |  |  |
| Total Effect | 0.043(0.018,0.067) | 0.024(0.001,0.046) |
| Direct Effect | 0.039(0.014,0.065) | 0.011(-0.014,0.034) |
| Mediated Proportion (%) | NA | 52.66(5.93,351.99) |
| Mediated Effect *P*-value | 0.376 | 0.046 |
| CRI-II |  |  |
| Total Effect | 0.024(0.001,0.048) | 0.023(0.001,0.047) |
| Direct Effect | 0.018(-0.006,0.044) | 0.018(-0.005,0.041) |
| Mediated Proportion (%) | NA | NA |
| Mediated Effect *P*-value | 0.164 | 0.172 |
| LCI |  |  |
| Total Effect | 0.056(0.033,0.081) | 0.021(-0.001,0.047) |
| Direct Effect | 0.054(0.030,0.081) | 0.008(-0.016,0.032) |
| Mediated Proportion (%) | NA | NA |
| Mediated Effect *P*-value | 0.528 | 0.056 |
| RC |  |  |
| Total Effect | 0.043(0.020,0.065) | 0.025(0.003,0.048) |
| Direct Effect | 0.040(0.017,0.063) | 0.016(-0.006,0.040) |
| Mediated Proportion (%) | NA | 35.62(8.37,156.11) |
| Mediated Effect *P*-value | 0.164 | 0.030 |
| RC/HDL-C |  |  |
| Total Effect | 0.060(0.035,0.085) | 0.024(0.001,0.047) |
| Direct Effect | 0.058(0.034,0.084) | 0.009(-0.013,0.033) |
| Mediated Proportion | NA | 61.57(21.51,329.67) |
| Mediated Effect *P*-value | 0.404 | 0.036 |
| Models were adjusted for age, sex, BMI, education level, marital status, smoking, drinking status, hypertension, and dyslipidemia drug. AIP, atherogenic index of plasma; LCI, lipoprotein combined Index; CRI-I, cardiovascular risk index-I; CRI-II, cardiovascular risk index-II; RC, remnant cholesterol; RC/HDL-C, remnant cholesterol/high density lipoprotein cholesterol; AC, atherogenic coefficient; Non-HDL-C, non- high density lipoprotein cholesterol. | | |


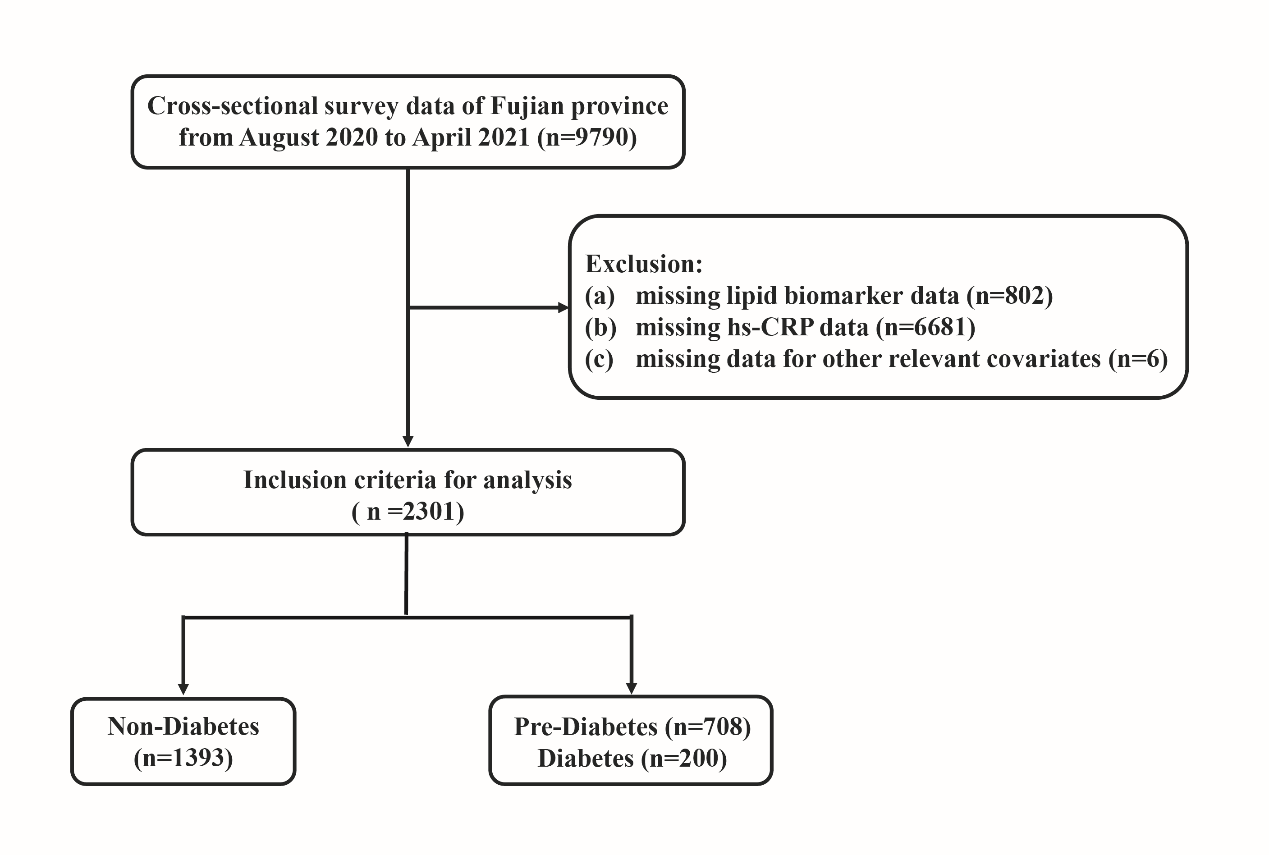


Figure S1 Participant inclusion and exclusion flowchart

**
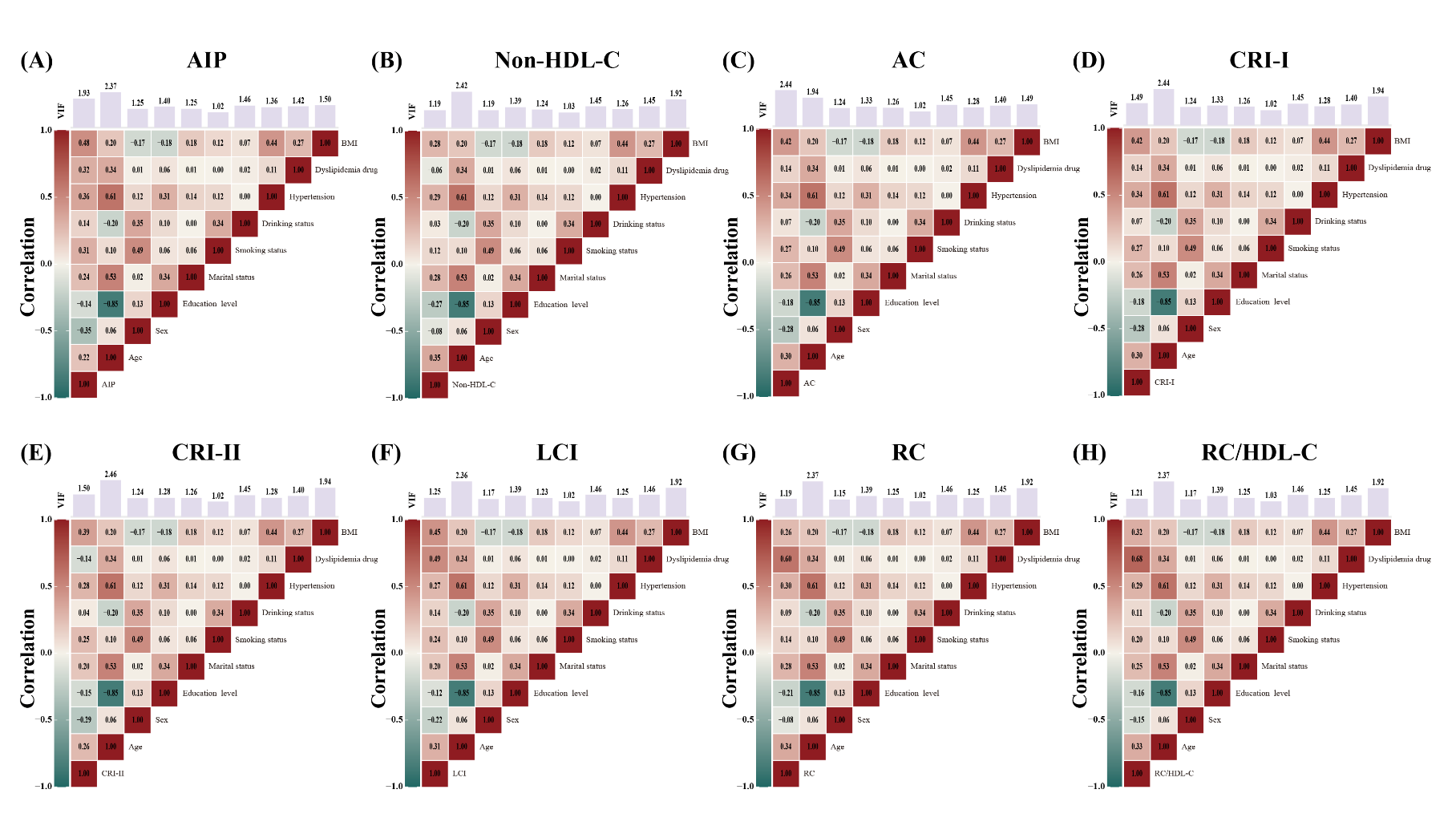
**

Figure S2 Correlation analysis and VIF assessment of non-traditional lipid parameters with covariates in the models. (A) AIP, atherogenic index of plasma, (B) Non-HDL-C, non- high density lipoprotein cholesterol, (C) AC, atherogenic coefficient, (D) CRI-I, cardiovascular risk index-I, (E) CRI-II, cardiovascular risk index-II, (F) LCI, lipoprotein combined Index, (G) RC, remnant cholesterol, (H) RC/HDL-C, remnant cholesterol/high density lipoprotein cholesterol.


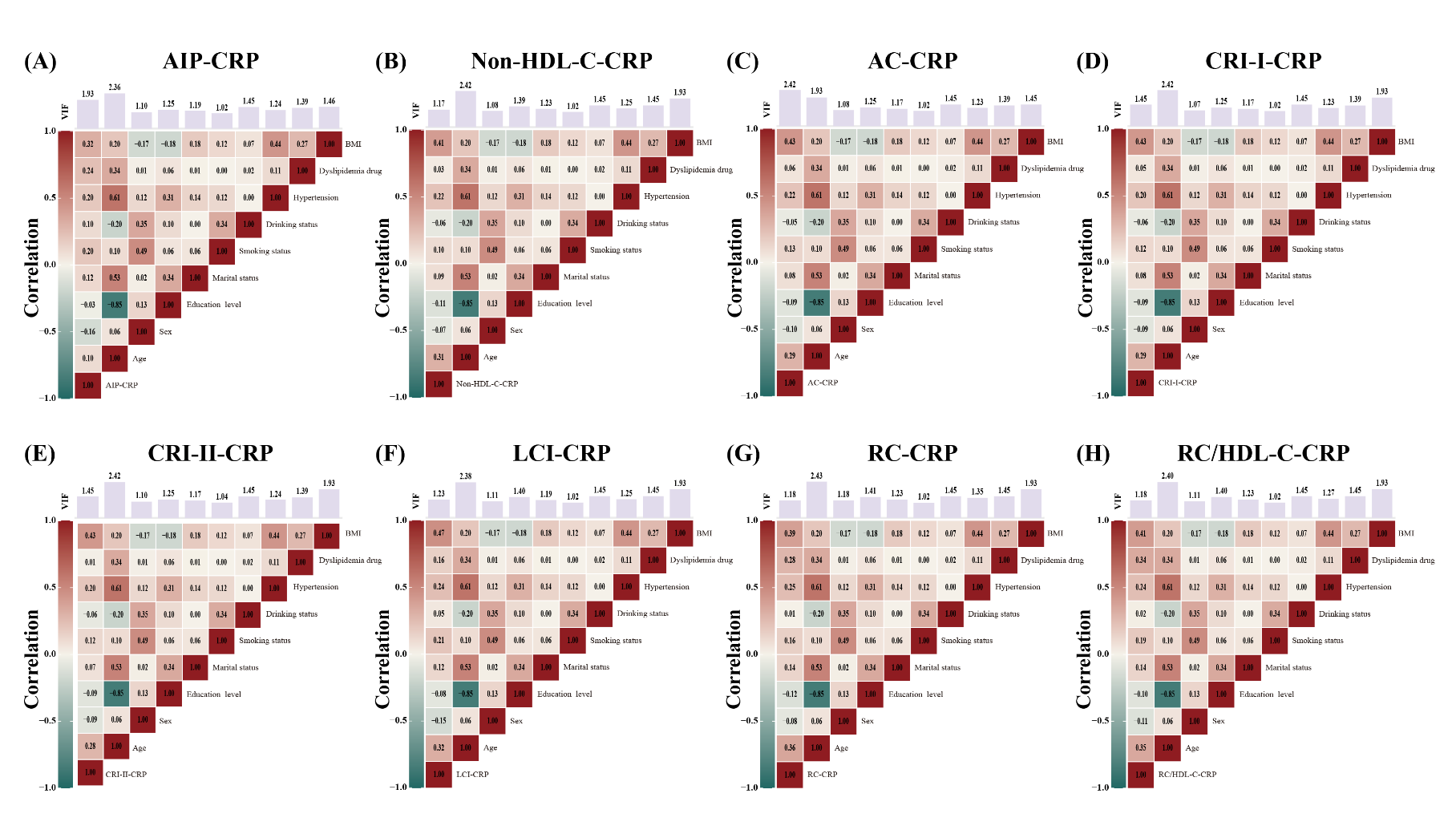
Figure S3 Correlation analysis and VIF assessment of non-traditional lipid parameters with covariates in the models. CRP refers to high-sensitivity C-reactive protein (hs-CRP) throughout this study. (A) AIP, atherogenic index of plasma, (B) Non-HDL-C, non- high density lipoprotein cholesterol, (C) AC, atherogenic coefficient, (D) CRI-I, cardiovascular risk index-I, (E) CRI-II, cardiovascular risk index-II, (F) LCI, lipoprotein combined Index, (G) RC, remnant cholesterol, (H) RC/HDL-C, remnant cholesterol/high density lipoprotein cholesterol.


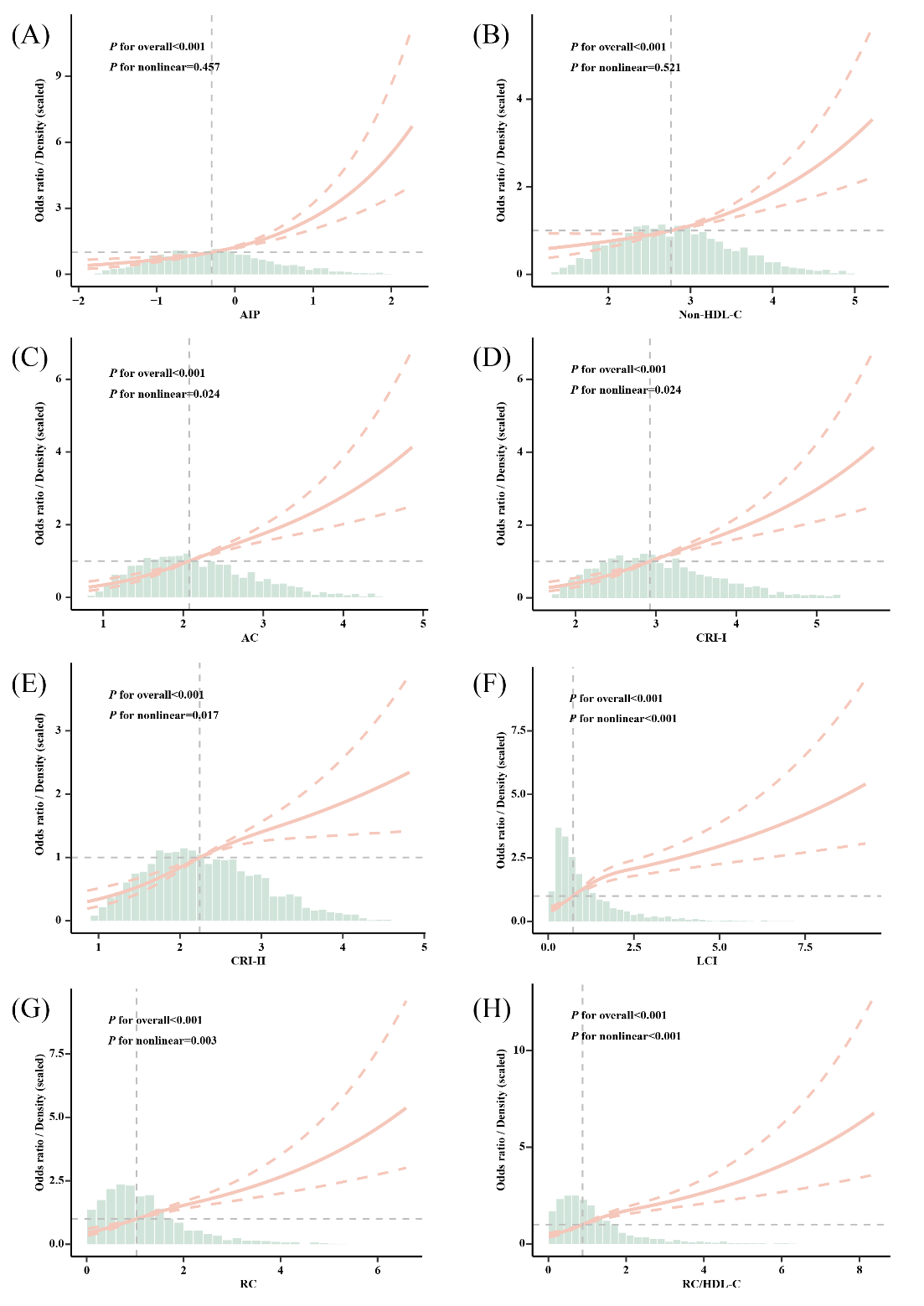


Figure S4 Dose-response relationship between non-traditional lipid parameters and diabetic status. Models were adjusted for age, sex, BMI, education level, marital status, smoking, drinking status, hypertension, and dyslipidemia drug. (A) AIP, atherogenic index of plasma, (B) Non-HDL-C, non- high density lipoprotein cholesterol, (C) AC, atherogenic coefficient, (D) CRI-I, cardiovascular risk index-I, (E) CRI-II, cardiovascular risk index-II, (F) LCI, lipoprotein combined Index, (G) RC, remnant cholesterol, (H) RC/HDL-C, remnant cholesterol/high density lipoprotein cholesterol.


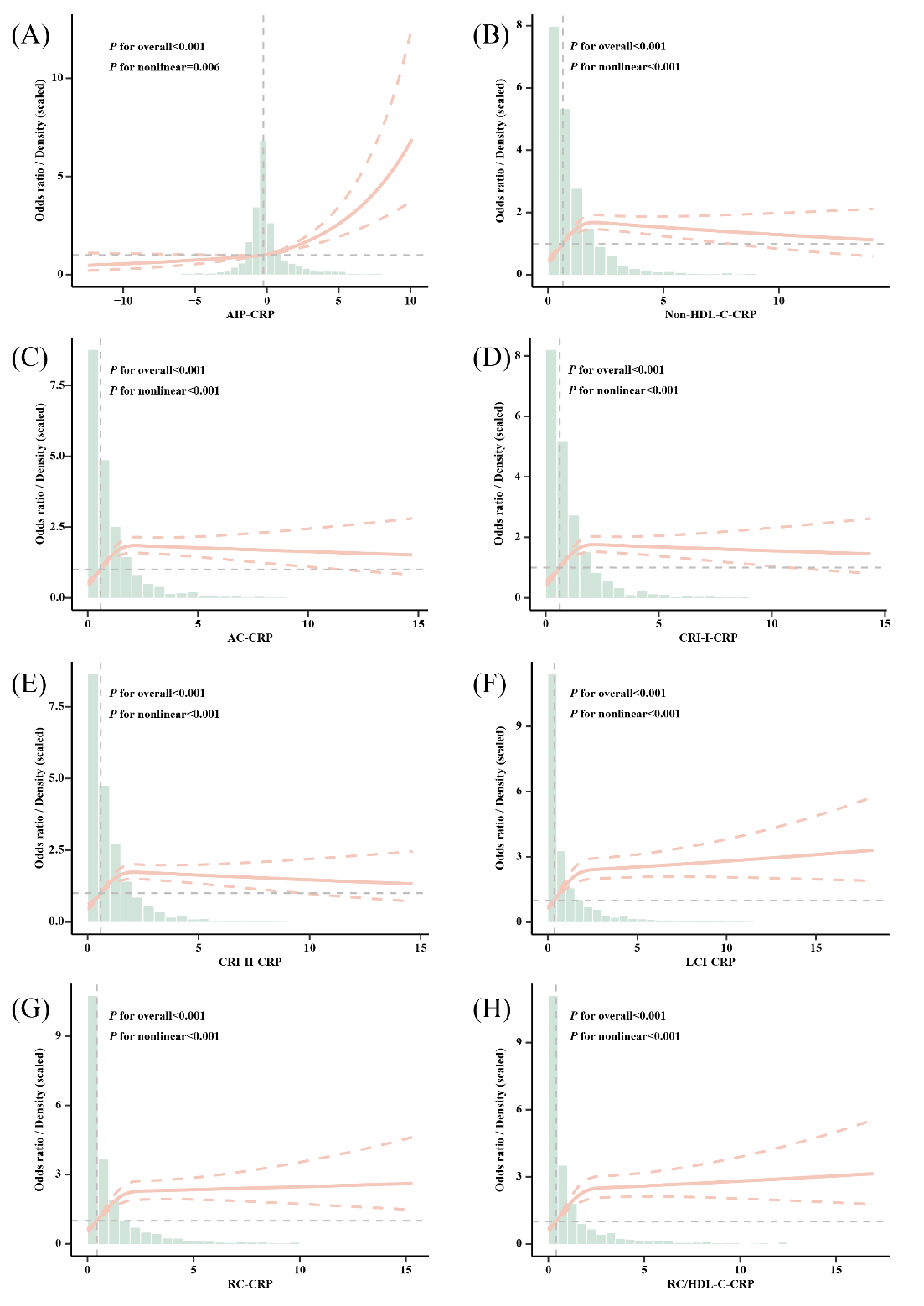
Figure S5 Dose-response relationship between non-traditional lipid-inflammatory parameters and diabetic status. Models were adjusted for age, sex, BMI, education level, marital status, smoking, drinking status, hypertension, and dyslipidemia drug. CRP refers to high-sensitivity C-reactive protein (hs-CRP) throughout this study. (A) AIP, atherogenic index of plasma, (B) Non-HDL-C, non- high density lipoprotein cholesterol, (C) AC, atherogenic coefficient, (D) CRI-I, cardiovascular risk index-I, (E) CRI-II, cardiovascular risk index-II, (F) LCI, lipoprotein combined Index, (G) RC, remnant cholesterol, (H) RC/HDL-C, remnant cholesterol/high density lipoprotein cholesterol.


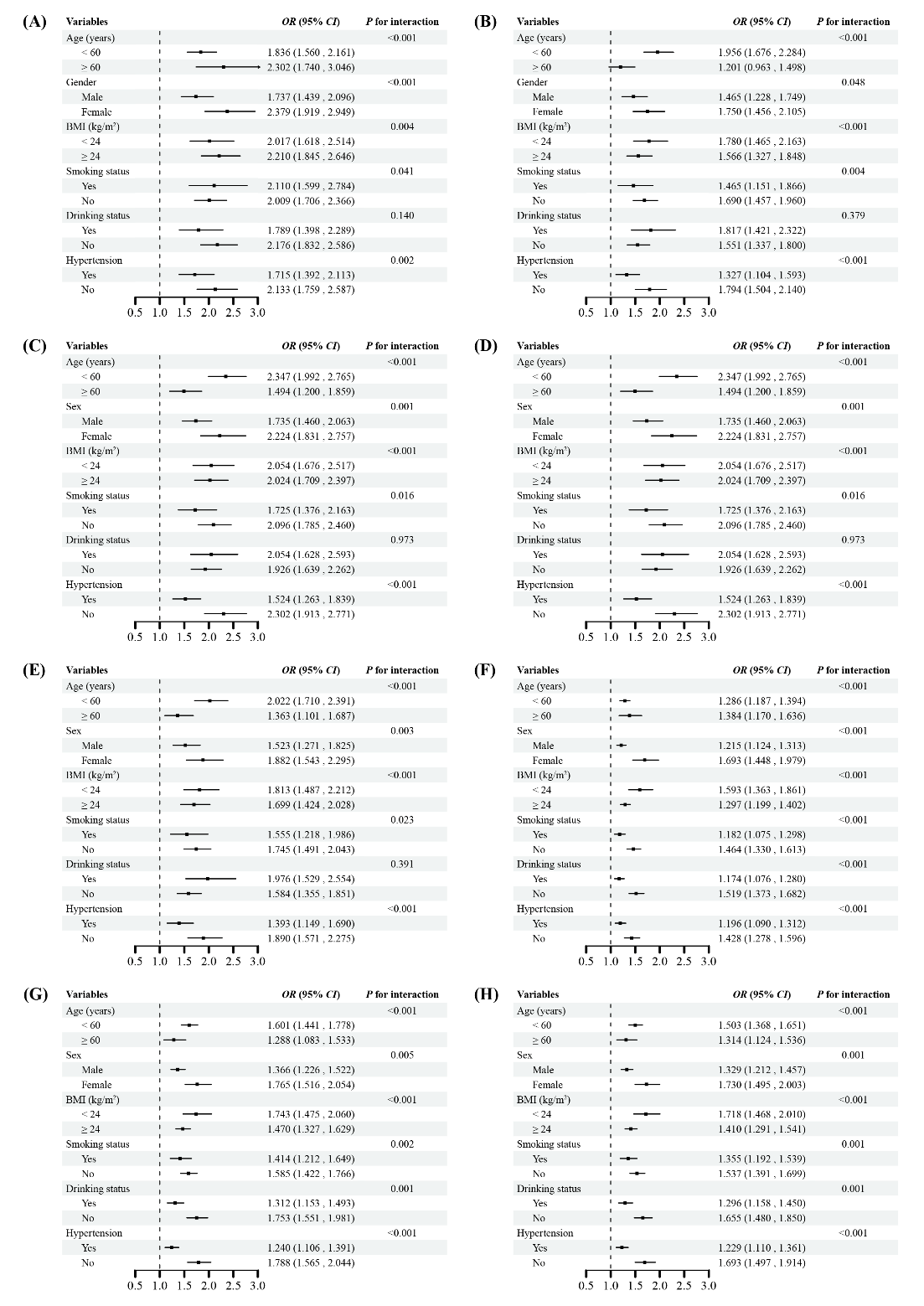


Figure S6 Forest plot of subgroup analysis on the association between non-traditional lipid parameters and diabetic status. Models were adjusted for age, sex, BMI, education level, marital status, smoking, drinking status, hypertension, and dyslipidemia drug. (A) AIP, atherogenic index of plasma, (B) Non-HDL-C, non- high density lipoprotein cholesterol, (C) AC, atherogenic coefficient, (D) CRI-I, cardiovascular risk index-I, (E) CRI-II, cardiovascular risk index-II, (F) LCI, lipoprotein combined Index, (G) RC, remnant cholesterol, (H) RC/HDL-C, remnant cholesterol/high density lipoprotein cholesterol.


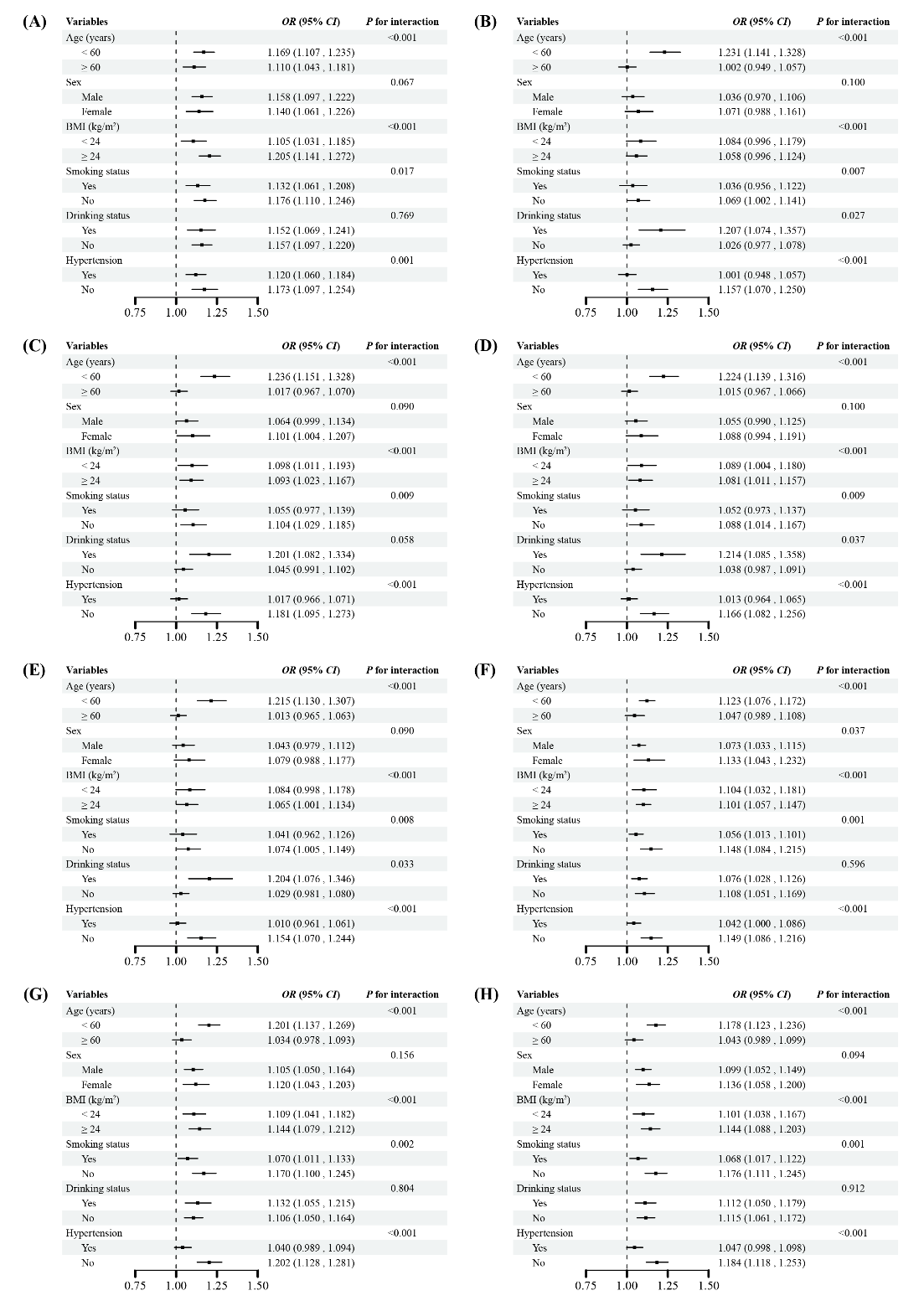
Figure S7 Forest plot of subgroup analysis on the association between non-traditional lipid-inflammatory parameters and diabetic status. Models were adjusted for age, sex, BMI, education level, marital status, smoking, drinking status, hypertension, and dyslipidemia drug. CRP refers to high-sensitivity C-reactive protein (hs-CRP) throughout this study. (A) AIP, atherogenic index of plasma, (B) Non-HDL-C, non- high density lipoprotein cholesterol, (C) AC, atherogenic coefficient, (D) CRI-I, cardiovascular risk index-I, (E) CRI-II, cardiovascular risk index-II, (F) LCI, lipoprotein combined Index, (G) RC, remnant cholesterol, (H) RC/HDL-C, remnant cholesterol/high density lipoprotein cholesterol.
